# Structural, Mechanical, and Environmental Assessment of a Poly(butylene adipate-*co*-terephthalate) (PBAT)-Inulin Composite Material

**DOI:** 10.1021/acssuschemeng.5c13077

**Published:** 2026-03-11

**Authors:** Francesco Brenda, Silvia Barbi, Monia Montorsi, Duccio Gallichi-Nottiani, Maria Grimaldi, Olimpia Pitirollo, Corrado Sciancalepore, Daniel Milanese, Daniele Cespi

**Affiliations:** † Department of Industrial Chemistry “Toso Montanari”, 9296 University of Bologna, Via Piero Gobetti, 85, Bologna 40129, Italy; ‡ Department of Science and Methods for Engineering, 9306 University of Modena and Reggio Emilia, Via Amendola 2, Reggio Emilia 42122, Italy; § Department of Systems Engineering and Industrial Technologies − DISTI, 9370 University of Parma, Parco Area delle Scienze 181/A, Parma 43124, Italy; ∥ National Interuniversity Consortium of Materials Science and Technology (INSTM), Via G. Giusti 9, Florence 50121, Italy; ⊥ Department of Chemistry, Life Science, and Environmental Sustainability − SCVSA, 9370 University of Parma, Parco Area delle Scienze 17/A, Parma 43124, Italy; # Interdepartmental Centre of Industrial Research “Renewable Resources, Environment, Sea and Energy”, University of Bologna, Via Dario Campana, 71, Rimini, Rimini 47922, Italy

**Keywords:** poly(butylene adipate-*co*-terephthalate), PBAT, inulin, composite, design of experiment, DoE, life cycle assessment, LCA

## Abstract

The growing interest in mitigating the effects associated with the extensive production and consumption of fossil-based plastics has led to increasing efforts in the development of biobased and biodegradable materials. In this setting, poly­(butylene adipate-*co*-terephthalate) (PBAT) has emerged as a viable biodegradable alternative to traditional polyesters. In this study, the manufacture of a PBAT-inulin composite film is investigated to assess its structural, mechanical, and environmental properties. A design of experiments (DoE) approach was applied to limit the number of experiments and find potential multivariate correlations (*p*-value <0.005) between composite formulation, e.g., inulin content, and mechanical properties. Results show that the inulin percentage has the highest influence on the strain at break, which is found to decrease as the percentage of inulin increases; as such, an ideal amount of inulin is found to be equal to 4.4−4.5% of the composite. From an environmental standpoint, results of a cradle-to-gate life cycle assessment (LCA) (1 kg of composite as the functional unit) show that PBAT production is the highest overall contributor to the impacts of the composite (68% average across categories), whereas inulin presents the highest contribution in the marine eutrophication (71%) and land-occupation (44%) categories. Among the processing steps, composite extrusion reports the highest average impacts at 14%. Also, a sensitivity analysis suggests that adopting biobased PBAT and increasing the percentage of renewable electricity consumed could reduce the cumulative environmental burdens. Overall, this integrated approach can provide valuable information for further optimization of both mechanical performance and environmental sustainability, in line with the principles of Green Chemistry and Green Engineering.

## Graphical abstract

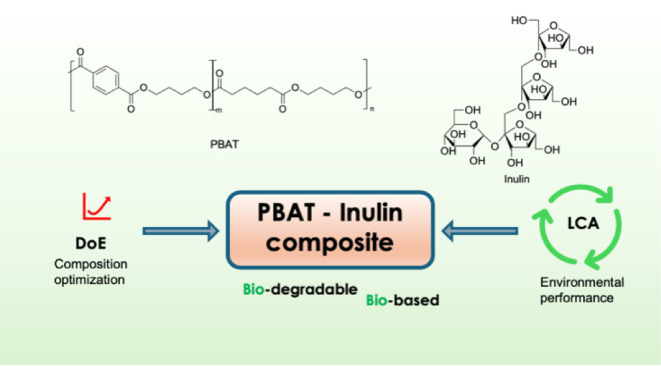

## Introduction

The widespread use of fossil-based plastics has been a major environmental concern because of their lingering presence in ecosystems and reliance on limited raw material supplies.^1^ Plastic pollution is receiving increasing attention,^2^ with particular focus on the pressing challenges presented by the release of micro and nanoplastics in the environment, reportedly associated with adverse toxic effects and potential human health risks.^3^ Reducing the share of fossil-based polymers in favor of biodegradable alternatives is a potential strategy to mitigate the issues associated with plastic pollution and microplastics release,^4^ although the environmental fate and degradation products, e.g., microbiodegradable plastics, still need further research.^5,6^ The increased market presence of biodegradable products is mainly driven by regulations aimed at reducing the overall production and consumption of short-lived single-use conventional plastics.^7^ In 2022, the annual global plastic production was equal to 400 Mt, 90% of which consisted of virgin plastic resins,^8^ mainly derived from fossil-based precursors. Current plastics production mainly relies on fossil resources both as feedstock and energy carriers,^9^ implying issues such as greenhouse gases (GHG) emissions (4.5% of global GHG emissions in 2015), resources depletion, and human health-related emissions of particulate matter.^10^ Given the trends showing an increase in the production of plastics, potentially reaching over 880 Mt per year in 2050,^11^ the most promising strategies to mitigate the environmental burdens associated with plastic production and consumption include improving the rates of chemical and mechanical recycling as well as using biomass and CO_2_ as feedstock.^12,13^ Hence, there is now great interest in the production of biodegradable, biobased products that are in line with the principles of sustainable development and circular economy.^14^ Among biodegradable materials, poly­(butylene adipate-*co*-terephthalate) (PBAT) plays an important strategic role, displaying mechanical properties comparable to those of conventional plastics like low-density polyethylene, but also possessing proven biodegradability in industrial composting conditions.^15^ PBAT is obtained by polycondensation reaction from different fossil-based monomers, butanediol (BDO), adipic acid (AA), and terephthalic acid (PTA). In 2022, the production of PBAT in China exceeded 1 million metric tons, representing 83% of global production.^16^ Even though PBAT has traditionally been made with petrochemical feedstocks, new innovations have allowed for the partial substitution of its monomers with renewably sourced alternatives, enhancing its environmental characteristics.^17,18^ In order to investigate the environmental performance of PBAT, life cycle assessment (LCA) is a standardized methodology under ISO 14040 and 14044 that allows to quantify the potential environmental impacts associated with a product, system, or service throughout its life cycle, i.e., from the extraction of raw materials to its end-of-life stage.^19,20^ In previously published literature, the environmental implications of the production of PBAT have been evaluated by means of LCA, focused on the production of its fossil-based monomers and their substitution with biobased alternatives.^16,21–24^ The production of PBAT in an industrial context has been investigated to compare the production of the fossil-based polyester with biobased routes. Cradle-to-gate LCAs based on process simulation and company data both report superior performances for the biobased alternative compared to the fossil counterpart.^16,21^ PBAT composites reinforced with inorganic fillers have also been evaluated from an environmental standpoint via LCA, with the aim of comparing the biodegradable composite with conventional solutions from a cradle-to-grave perspective.^23,24^ Zhou et al. present a cradle-to-gate LCA of a PBAT composite film reinforced with montmorillonite and lignin, comparing its environmental performance against pure PBAT and polyethylene films. Further context and literature review are provided in the Supporting Information. More innovations in PBAT performance, cost-effectiveness, and life-cycle impacts are, however, needed to expand usability, particularly in sectors such as packaging and agriculture. A viable method is the incorporation of natural fillers from renewable or waste-derived sources, enhancing the material’s functionality while decreasing dependence on virgin material. Inulin, a plant-derived polysaccharide extracted, among others, from chicory or Jerusalem artichoke, is also a good biofiller for composite products. The substance is composed of oligo- and polysaccharides with different chain lengths and is heavily used in the food sector.^25^ Thus, it represents a valid alternative for a more sustainable material production. Interestingly, the addition of inulin in PBAT films offers the opportunity of studying the mechanical and barrier performances of an innovative material, while potentially affecting its environmental impact.^26^ The inherent variability of inulin’s composition, however, necessitates exhaustive characterization to ensure consistency and maximize the composite’s performance. The use of analytical techniques such as high-performance anion exchange chromatography coupled with pulsed amperometric detection (HPAEC-PAD) allowed for the accurate quantification of the inulin’s molecular composition, thus enabling its effective incorporation in polymer matrices.^25^ Our study investigates the production of PBAT−inulin biopolymer composite films, highlighting their structural, mechanical and environmental characteristics under an integrated approach. In using inulin from renewable sources and upgrading agricultural food waste, the study proposes standardized solutions for the production of a biobased composite material that has high levels of environmental sustainability and performance. As a first innovative aspect in comparison with the consolidated literature, and in order to limit the number of experiments and produce mathematical models that can forecast correlations between mechanical properties and the material formulation, the formulation selected for this study was then generated using a Design of Experiments (DoE) approach. Furthermore, the LCA methodology was applied to investigate the impacts associated with the manufacture of PBAT-inulin composite, to integrate mechanical and statistical analyses with environmental considerations at early stages. The present work introduces a novel biocomposite based on Poly­(butylene adipate-*co*-terephthalate) (PBAT) and inulin, distinguishing itself from existing literature through the strategic selection of a filler capable of modulating biodegradation kinetics and the implementation of a solvent-free fabrication protocol.^6^ Unlike other fillers, such as lignin, which frequently requires organic solvents for effective incorporation or modification, our approach eliminates these reagents. While lignin often acts as a stabilizer, inulin may act as a sacrificial phase or a nutrient source for specific microbial consortia, thereby potentially accelerating the disintegration rate in composting environments compared to lignin-stabilized blends. Methodologies recently reported, such as those in Hasan et al. (2025), often rely on chemical pretreatments or compatibilization strategies involving organic solvents or synthetic modifiers to bridge the polarity gap between the hydrophilic filler and the hydrophobic PBAT matrix. In contrast, our study adopts a strictly solvent-free methodology.^24^ By leveraging melt-processing techniques, we also eliminate the emission of Volatile Organic Compounds (VOCs). The omission of solvent recovery stages not only optimizes the Life Cycle Assessment (LCA) profile but also significantly enhances the industrial scalability of the inulin-PBAT system. Compared to the existing literature, in this study, the adoption of an integrated approach of DoE and LCA allows to individuate an optimal composite formulation, highlighting potential mechanical advantages and limitations of reinforcing PBAT with an inulin biobased filler and identifying its environmental hotspots. Moreover, conducting the analysis at laboratory scale allows to inform a more sustainable technological upscaling, as at this stage developers have the highest degrees of freedom for material and process optimization. In supporting the development of a biodegradable composite material and the integration of biobased renewable feedstock in its formulation, the work is in line with the principles of Green Chemistry and Green Engineering.^27,28^

## Materials and Methods

Commercial PBAT granules, purchased by MAgMa Spa (Italy), were subjected to cryogenic milling to reduce them to a fine polymer powder, which was subsequently dried in a static oven at 40 °C for 12 h to remove residual moisture. Two different types of inulin were used as fillers: inulin D, characterized by a shorter chain length, i.e., low degree of polymerization (DP), DP­(max) = 20, and inulin T, with a longer chain length, i.e., higher degree of polymerization, DP­(max) = 60. Prior to processing, both inulin types were sieved to obtain a controlled particle size distribution ranging between 50 and 150 μm and then dried under the same conditions (40 °C, 12 h) to minimize moisture-related degradation during melt processing.

Dried powders of PBAT and inulin were mixed at different filler concentrations (5 and 10% w/w), as specified in the experimental plan. Mixture powders were fed into a corotating twin-screw extruder under optimized conditions to achieve homogeneous dispersion of the inulin in the polymer matrix. Extrudates obtained were pelletized and further processed in an injection molding machine with a mold suitable to obtain Type 5A dumbbell specimens, following ISO 527-2:2012 standard for tensile testing.^29^

The mechanical properties of the obtained PBAT-inulin composite are discussed in the “Considerations on the Mechanical Properties of the Composite” section, reported in the Supporting Information.

Specific compositions and sample codes of the prepared composites—namely, PBAT with inulin D and T at both concentration levels—are tabulated in Table 1.

**Table 1:** Experimental Plan of the Prepared PBAT Composite Materials

Compositions *ID*	PBAT *wt %*	Inulin D *wt %*	Inulin T *wt %*
*PBAT*	100	0	0
PBAT_D5	95	5	0
PBAT_D10	90	10	0
PBAT_T5	95	0	5
PBAT_T10	90	0	10

### Statistical Analysis

To increase the effectiveness of the mechanical properties in terms of the quantity and type of inulin, a DoE approach was used. By overcoming the significant simplifications inherent in the so-called One-Factor-at-A-Time (OFAT) method, DoE saves time and money by lowering the number of experiments required to get the most information possible on complicated situations.^30,31^ The trial was carried out with ten replicates for each formulation, facilitating the assessment of the model’s lack of fit and its overall reliability. The experiments were executed following a randomized run order to prevent any environmental bias. To address some of the inherent limitations of this approach (such as restrictions to combinations at the edges of the area of interest), an augmented 2-level full factorial design model was chosen including central points among lowest and highest level. Therefore, the Computer Aided D-optimal Design was applied to control the experimental procedure.^31^ A total of 50 experiments, including center points and repetition were designed. Input factors and responses are reported in Tables 2 and 3, whereas in Table S1 the complete experimental plan has been presented including all the results at specific measurement points.

**Table 2:** Input Factors and Their Level for the Experimental Plan Design

Factor	Name	Units	Type	Subtype	Lowest level	Highest level	Goal	Importance
A	Inulin type		Categoric	Nominal	D	T	In range	3
B	Quantity	%	Numeric	Continuous	0.00	10.00	Maximize	4

The analysis of variance (ANOVA) through F-test was utilized to assess the significance of the model and its ability to predict the relationships between compositions and properties. With F-test is possible to evaluate variances or to assess the significance of a regression model by comparing the explained variance to the unexplained variance. The resulting F-statistic follows an F-distribution, and the associated p-value indicates whether the observed variance ratio is statistically significant. In the present study p-values exceeding 0.005 were deemed not statistically significant and were therefore excluded from the model equation.^32^ The quality of the fit, in terms of regression analysis, and the predictive capability of the models were assessed using lack of fit test, Adj-R^2^ and Pred-R^2^. Lack of fit (LoF) test is a statistical procedure used to assess whether a chosen model adequately describes the relationship between the independent factors and the response variable. LoF test compares the variation of the experimental data that is not explained by the model (residual error) with the pure experimental error (replication error). Thereafter, a significant lack of fit indicates that the model may be inappropriate, suggesting that the relationship between factors and response is not fully captured. In strong similarity with the F-test, an associated p-value exceeding 0.005 was considered as a not significant lack of fit, meaning the model adequately describes the data Adj-R^2^ represents the proportion of variance in the dependent variables that can be explained by the independent variables (also by considering the total degree of freedom of the investigated system), while Pred-R^2^, which is calculated from the predicted values of the dependent variables, serves a similar purpose.^33^ The Box−Cox Plot diagnostic tool was employed to determine whether a mathematical transformation was necessary for the response data to address issues related to low fitting due to data magnitude. Additionally, the response interaction plot was utilized as a functional tool to illustrate the influence of the main components on the final property, effectively presenting the key findings derived from the ANOVA. Experimental plan assessment and data analysis were performed using Design Expert software (version 13, Stat-Ease).

All the significant obtained models were integrated into the desirability function (D), which encompasses objectives and their importance, to derive a unique material formulation that meets all requirements. The overall desirability function yields the most favorable response values by considering all analyzed responses simultaneously.^34^ Each response is weighted based on its specific objective and importance, reflecting the extent to which it aligns with the defined purpose, and is subsequently averaged. The objective (or goal) specifies the desired direction or target for a response variable. It indicates whether the response should be maximized, minimized, or set to a specific target value. Importance represents the relative weight assigned to a response variable in the overall desirability function. It reflects how critical that response is compared to others when combining multiple responses into a single overall desirability score. Normally the value for the lowest importance is set equal to 1, whereas the value for the highest importance is set to 5. Objectives (or goals) and importances are defined by the analyst depending on constrains or real-world requirements. The desirability function value ranges from 0 to 1, where a value of 0 signifies a completely undesirable combination of independent factors, while a value of 1 denotes a fully desirable or optimal combination. In the present study, notably, the highest importance level (equal to 5) was assigned to stress at break, with the objective of maximizing this factor (Table 3). To inulin quantity was assigned a significance level of 4, with the aim of maximizing it as well (Table 2).

**Table 3:** Response Variables and Their Goal and Importance

Response	Units	Goal	Importance
*Stress at break*	MPa	In range	5
*Strain at break*	%	Maximize	3
*Young Module*	MPa	In range	3

### Life Cycle Assessment

Life Cycle Assessment (LCA) methodology was utilized to evaluate the potential environmental impacts of synthesizing the PBAT:inulin (95:5) composite at laboratory scale.

LCA is a standardized approach,^19,20^ recognized worldwide as one of the principal tools to address the environmental sustainability of products, processes, and systems. The analysis focused on a cradle-to-gate system boundaries (Figure 1), encompassing all stages up to the production of 1 kg of composite, which served as the functional unit (FU).

The ReCiPe 2016 (H), v 1.11 method was applied to consider different impact at midpoint level,^35^ such as GWP: Global warming (kg CO_2_ eq); ODP, Stratospheric ozone depletion (kg CFC11 equiv); IRP, Ionizing radiation (kBq Co-60 equiv); HOFP, Ozone formation-human health (kg NOx eq); PMFP, Fine particulate matter formation (kg PM 2.5eq); EOFP, Ozone formation - terrestrial ecosystems (kg NOx eq); TAP, Terrestrial acidification (kg SO_2_ eq); FEP, Freshwater eutrophic. (kg P eq); MEP, Marine eutrophic. (kg N eq); TETP, Terrestrial ecotoxicity (kg 1,4-DCB eq); FETP, Freshwater ecotoxicity (kg 1,4-DCB eq); METP, Marine ecotoxicity (kg 1,4-DCB eq); HTPc, Human carcinogenic toxicity (kg 1,4-DCB eq); HTPnc, Human noncarcinogenic toxicity (kg 1,4-DCB eq); LOP, Land use occupation (m^2^a crop eq); SOP, Mineral resource scarcity (kg Cu eq); FFP, Fossil resource scarcity (kg oil eq); and WCP, Water consumption (m^3^). Results at end point level were also assessed to evaluate damages at the receptors level: human health, resources consumption, and ecosystem quality. In addition, the method developed by the Intergovernmental Panel on Climate Change (IPCC 2021, GWP100 incl. CO_2_ uptake, v.1.01) was selected to investigate more in depth the impacts on the climate change potential, since it can address the results in terms of carbon footprint.^36,37^

A baseline scenario, from this point forward indicated with the abbreviation “fossil-PBAT_S”, assumed the main precursors are produced from fossil resources (PBAT) and from chicory root (inulin). Primary data were collected to compile the foreground system, including the quantity and type of material used, as well as the waste and emission flows involved at each stage that characterize the system. Energy flows were not measured directly but were quantified based on appliance power (W) and usage time (h), using the same approach reported in literature.^38,39^ The Italian average mix was adopted. Supporting Information collects the whole inventory (Table S3). ecoinvent database (v3.10) and Agri-footprint were used to cover background information.^40,41^ Average market scenarios for Europe were selected when available, using the APOS U version (at point of substitution unit process).

Three alternative 95:5 composition sensitivity scenarios were also created to evaluate how the entire system is affected by variables such as the use of a portion of renewable energy (indicated as 50%PV_S), the use of biobased PBAT (indicated as bio-PBAT_S), and a combination of both (indicated as 50%PV + bio-PBAT_S). Full inventories are reported in the (Supporting Information Tables S3−S8). Further assessments were also performed to analyze the influence of the material composition (i.e., inulin quantity) on the environmental impacts of the composite, based on the tested compositions reported in section Statistical Analysis. In particular, a composition of 90:10 PBAT:inulin (10%Inulin_S) was considered, assuming that energy consumption to conduct the processing steps of matrix and reinforcement is linearly dependent on the mass of PBAT and inulin (i.e., PBAT and inulin drying, PBAT milling, and inulin sieving). Environmental performances were assessed both on a midpoint and an end point level, adopting the ReCiPe 2016 method.

Data quality was evaluated by the application of the pedigree matrix.^42^ Finally, a Monte Carlo analysis was performed assuming a log-normal distribution and an iteration of 1,000 runs.

**Figure 1:**
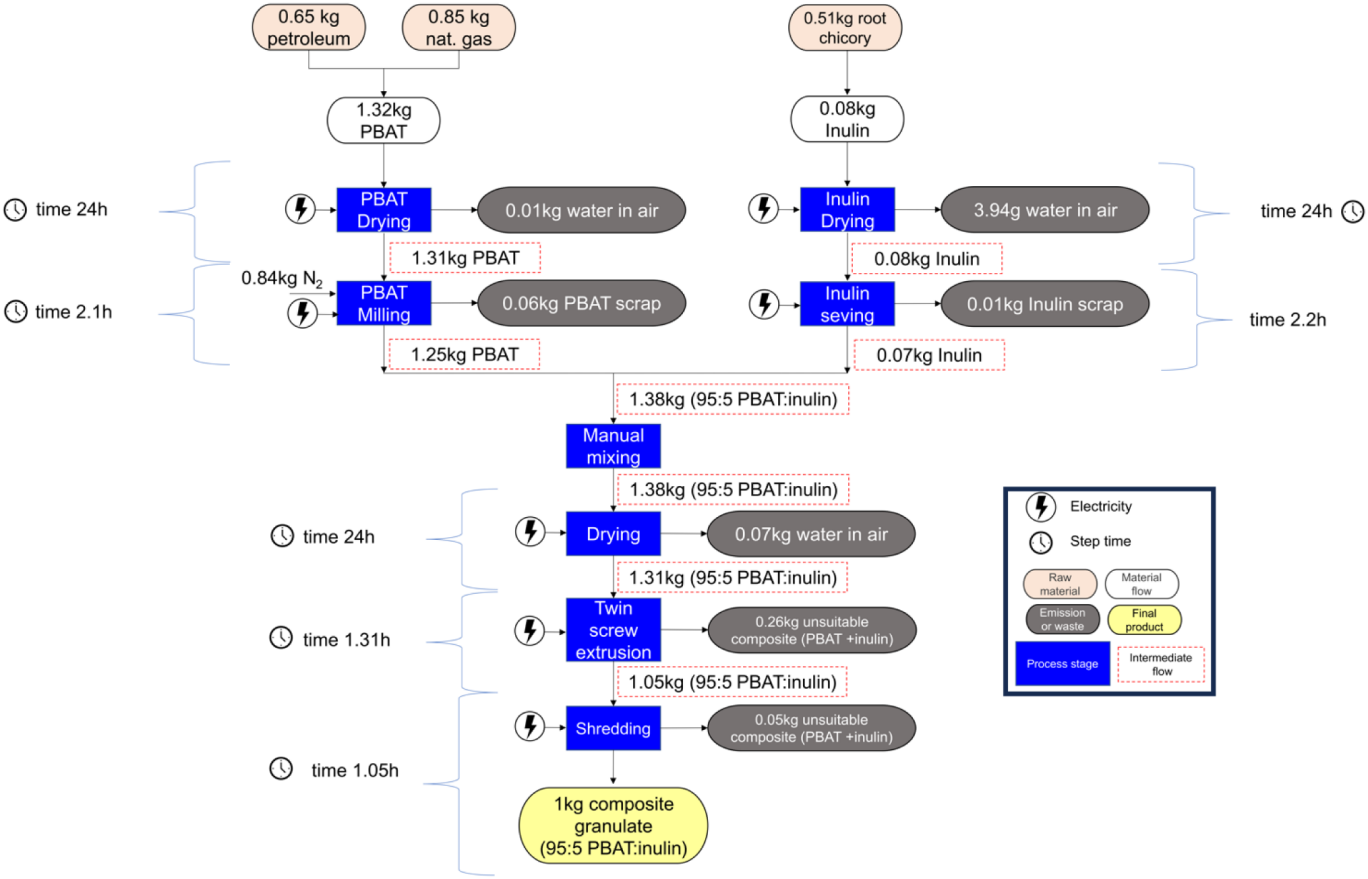
Cradle-to-gate system boundaries of the LCA study, including all life cycle stages from the extraction of raw materials up to the production of the composite at laboratory scale.

## Results and Discussion

### Multivariate Analysis

The ANOVA results (Table S9) show that all models are significant (F-test p-value <0.005 and Lack of Fit p-value >0.005) and that the fittings of the data on the calculated models are very good as the Adj-R^2^ and Pred R^2^ are all above 0.85. Therefore, the model equations shown in Table 4 can be used to predict the mechanical properties of the materials studied as the type and amount of inulin change within the ranges identified in Table 2. Figure 2, on the other hand, shows the relative trends for the coded coefficients of each variable to identify whether and in what terms a given variable has an influence or not on each response. The coded equation’s coefficients are useful for identifying the relative impact of the factors by comparing their value. In particular, from Figure 2 it is evident that the amount of inulin rather than its type plays a key role in determining mechanical properties. Of these, the most affected is Strain at break, which is found to decrease as the amount of inulin increases (by virtue of the negative sign of the coefficient). Stress at Break, on the other hand, results in the property least affected by the type and amount of inulin, with coefficients all very close to zero. Finally, Young’s Modulus is in an intermediate situation, also in agreement with its definition, as it depends on the stress/strain linear slope. This property turns out to be intermediately affected by the amount of inulin, also in interaction with the type of inulin. Contrary to what we have seen for the other two properties, in this case, the sign of the coefficient being positive, we observe an increase in Young’s modulus, hence in the stiffness of the material as the inulin content increases. In addition, it must be considered that a significant synergic effect among the type and quantity of inulin arises by estimating Young’s modulus. Mathematical models are also graphically expressed with the interaction plots from Figures S1−S3, clearly showing the restrained errors on the measured data employed for the model, leading to statistically significant trends.

**Figure 2:**
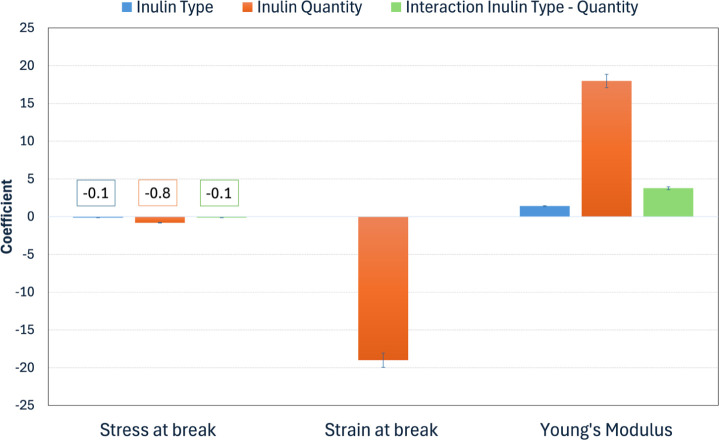
Coefficients of the coded equation for each response.

Notably, the desirability function (Table 4) suggests, in agreement with model results, that any type of inulin can be chosen without significantly impacting the mechanical properties of the materials. While an optimal amount of inulin is represented by a quantity value of 4.4−4.5%, as all formulations proposed and having these amounts of inulin, all have the same desirability value. It must be noted that, considering the definition of desirability function, the values obtained are average because they are close to the value between the maximum (1) and minimum (0) values that the desirability function can assume. The fact that high desirability values, close to 1, are not achieved confirms the significant challenge of maintaining mechanical properties in line with those of PBAT, while including high quantities of inulin.

**Table 4:** Desirability Function Results

Solution	Inulin type	Inulin quantity (%)	Stress at break (%)	Strain at break (%)	Young module (MPa)	Desirability(−)
1	D	4.539	8.632	88.498	139.710	0.446
2	T	4.539	8.412	88.496	138.970	0.446
3	D	4.414	8.649	90.130	139.354	0.446

### Life-Cycle Assessment Results

The impacts of the composite production for fossil-PBAT_S case were first investigated on a midpoint level, to evaluate the contribution of the production of PBAT, inulin, and the following processing steps to the overall burdens across categories. Complete results are reported in Figure 3 and Table S10. PBAT production emerges as the highest contributor across all categories, with an average impact equal to 68%, followed by the electricity consumed for the manufacture of the final PBAT-Inulin 95:5 composite. Among the processing steps, the twin-screw extrusion, air cooling, and winding of the composite report the highest electricity consumption at 3.90 kWh/FU, entailing the most elevated impacts among all process steps and reporting an average contribution of 14% across categories. Regarding the biobased reinforcement of the composite, inulin production results into the most significant contribution to the MEP (71%) and LOP (44%) categories (see Figures S4 and S5), mainly due to the dedicated cultivation of root chicory, despite inulin only accounting for 5% of the weight of the composite; process contributions for MEP and LOP categories are depicted in Figures S4 and S5, Supporting Information. As the main contribution to eutrophication and land use categories is associated with the upstream cultivation of chicory roots (inulin’s source), potential strategies to mitigate the impacts of the biobased fraction involve the valorization of chicory root byproducts (i.e., cascade biomass valorization strategies) and of inulin extraction from selected biowaste substrates, containing terpenes and polyphenols.^43,44^

Contributions above 20% are also encountered for TAP (20%) and HTPnc (23%). For the IRP category, the processing steps carry 52% of the overall burdens, due to electricity usage and the consumption of N_2_, used as inert gas in the PBAT milling step.

**Figure 3:**
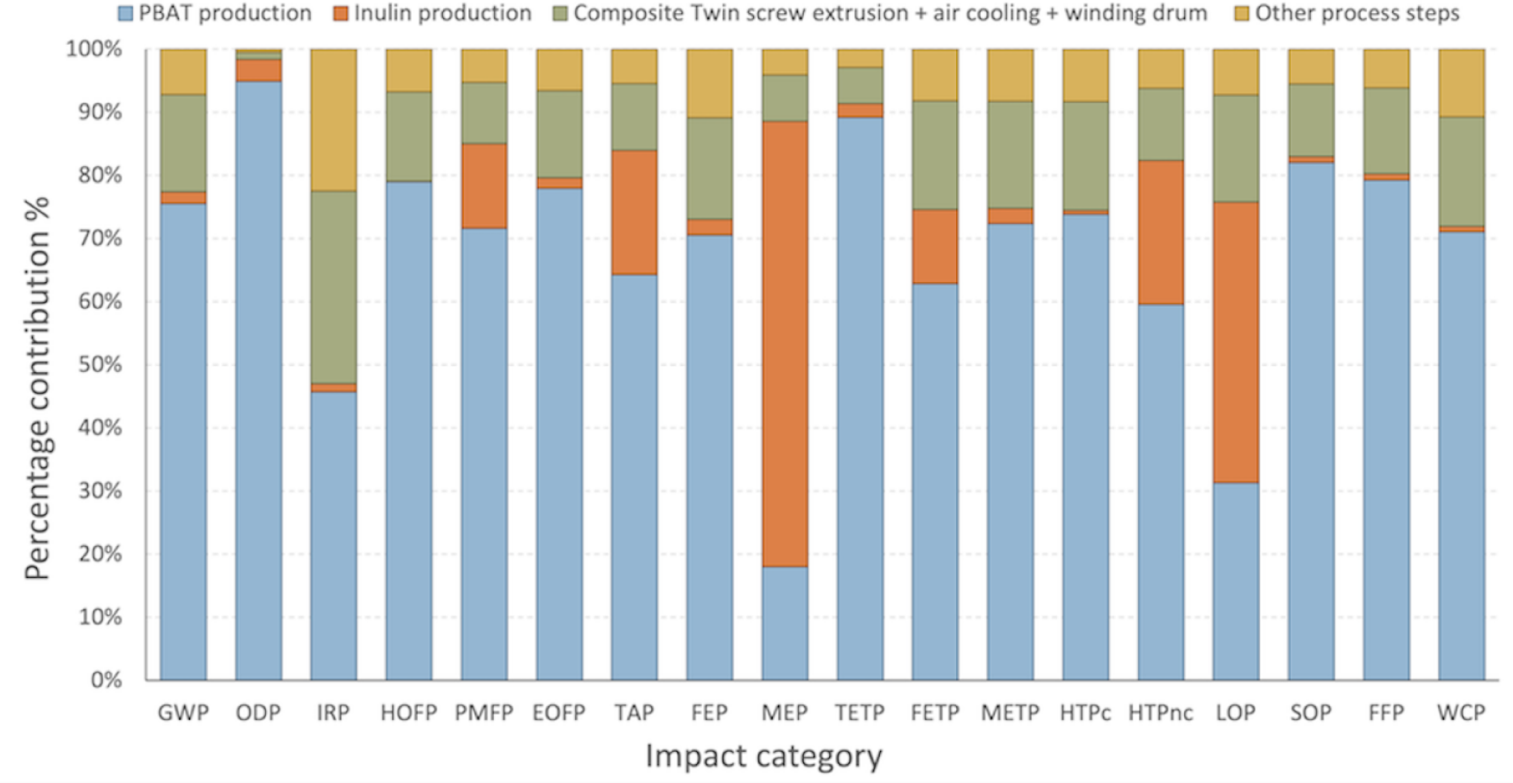
Contribution analysis on the midpoint environmental impacts for the production of the PBAT:inulin 95:5 composite, scenario fossil-PBAT_S; method: ReCiPe 2016 midpoint (H) v1.11/World 2010 (H).

The evaluation of midpoint results was then extended to 50%PV_S, bio-PBAT_S, and 50%PV + bio-PBAT_S. Results are reported in Table S10. 50%PV_S entails slight reductions compared to the baseline, due to the limited contribution of the composite processing steps to the final burdens; the highest reduction is equal to 18% in the IRP category, caused by the lower consumption of imported electricity from nuclear. Bio-PBAT_S constituted a potential 21% reduction in GWP and a 26% decrease in ODP impacts, associated with the substitution of fossil monomers with biobased ones. On the other hand, the impacts for MEP in bio-PBAT_S are 6.5 times higher than in fossil-PBAT_S. A similar trend is observed in the LOP category, with an impact 5.5 times higher than in fossil-PBAT_S. This highlighted the elevated contribution of biobased feedstock from dedicated cropland to these two categories, as also reported for inulin production in fossil-PBAT_S. 50%PV + bio-PBAT_S, integrating biobased precursors with the consumption of electricity at an increased renewable penetration, further accentuates the reduction of impacts in GWP and ODP categories, while mitigating the increase in MEP and LOP issues. Percentage results are reported in Figure S6.

On the end point categories, scenarios were compared based on potential damages associated with human health, ecosystems, and resources. Contributions were evaluated to produce PBAT and inulin as well as the subsequent operations to manufacture the final composite in fossil-PBAT_S case. Complete results are reported in Table S11. For fossil-PBAT_S, PBAT production composite accounts for 74% of damages on average across the evaluated categories (Figure S7), bearing the highest impacts on the resource scarcity issue. Alternative scenarios are also assessed, with 50%PV_S reporting slight reductions (−3% to −7%) for all three categories. The scenarios bio-PBAT_S and 50%PV + bio-PBAT_S also entail comparable impacts to fossil-PBAT_S in Human Health, reporting higher potential damages associated with Ecosystems (+52% and +46%, respectively) while also obtaining more significant reductions in the consumption of resources (−24% and −31% respectively) associated with the increased presence of biobased feedstock embodied in the final composite. Percentage results are summarized in Figure 4.

**Figure 4:**
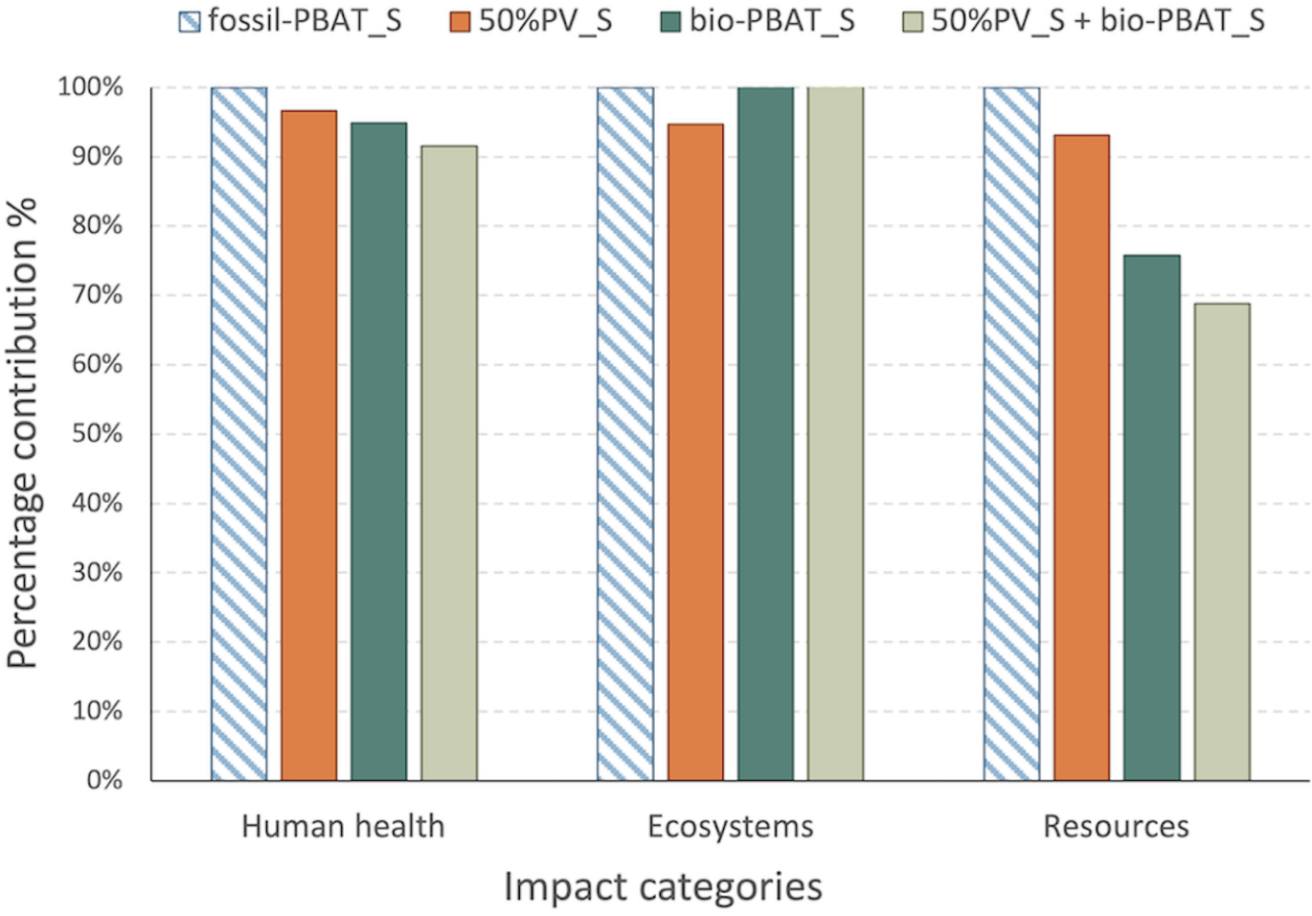
Comparative end point results for the assessed PBAT:Inulin 95:5 production scenarios, percentage end point impacts of 50%PV_S, bio-PBAT_S and 50%PV + bio-PBAT_S compared to fossil-PBAT_S (for Ecosystems, bio-PBAT_S and 50%PV + bio_PBAT_S impacts are equal to 152% and 146%, respectively), FU = 1 kg of composite; method: ReCiPe 2016 end point (H) v1.11/World 2010 (H/A).

Shifting the focus to the composition of the material, the comparison between fossil-PBAT_S and 10%Inulin_S at the midpoint level show that increasing the percentage of inulin to 10% of the composite weight would result into a significant increase in impacts for MEP (+62%) and LOP (+39%) categories, where the production of inulin reported the highest contribution on the overall impacts of the composite. Results are reported in Figure S8 and Table S12. On an end point level, after weighing, results for 10%Inulin_S differed less than 10% on an absolute value compared to fossil-PBAT_S, confirming the limited influence of the composition on the environmental performance of the composite for the considered range of reinforcement percentage. Results are reported in Table S13.

A detailed insight into the carbon footprint (CF) was also provided using the IPCC 2021. The method was employed to understand the contribution of fossil and biogenic carbon and of CO_2_ uptake to the cumulative global warming potential. Complete results are disclosed in Table S14. fossil-PBAT_S scenario presents the highest carbon footprint (13.2 kg CO_2_ eq.), mainly associated with the production of PBAT (74%, Figure S9), followed by 50%PV_S (12.3 kg CO_2_ eq.). For these scenarios, fossil CO_2_ eq. represents most of the overall carbon emissions of the process (96% and 97%). The bio-PBAT_S and 50%PV + bio-PBAT_S scenarios result in lower overall emissions compared to the other cases (11.2 and 10.3 kg CO_2_ eq., respectively). Both scenarios show an increase in biogenic CO_2_ emissions (4.2 and 4.0 kg CO_2_ eq.) and a corresponding CO_2_ uptake of −3.1 kg CO_2_ eq., consistent with the increased use of biobased materials.

Additional comments on a focus analysis conducted to evaluate the performance of PBAT production from fossil or biobased feedstock are reported in the (Supporting Information Figure S10, Tables S13−S14). Results are depicted in Figure 5.

**Figure 5:**
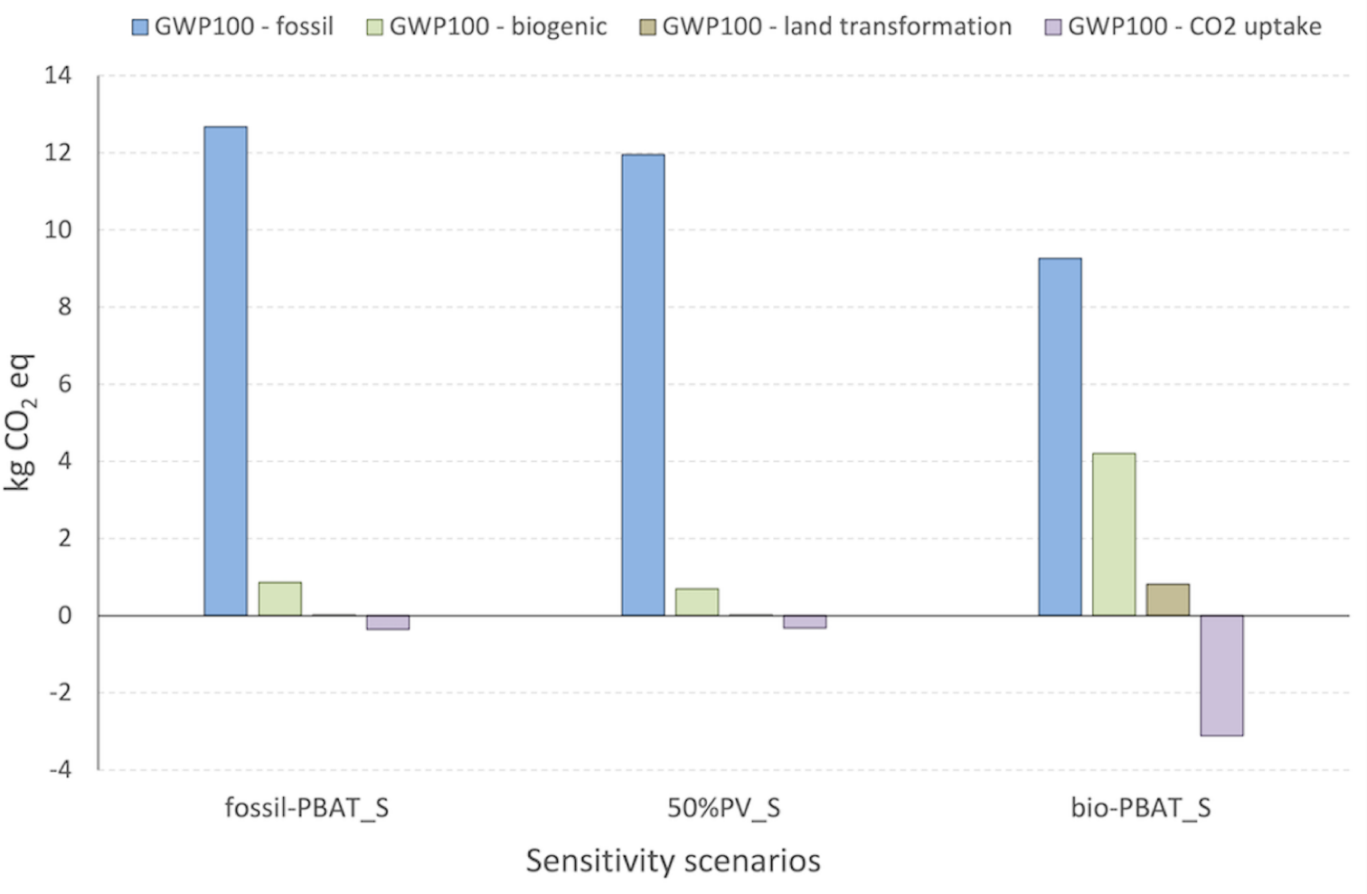
Carbon footprint of the production of the PBAT:Inulin 95:5 composite, calculated results for scenarios fossil-PBAT_S, 50%PV_S, bio-PBAT_S, 50%PV + bio-PBAT_S; method: IPCC 2021, GWP100 incl. CO_2_ uptake, v.1.01.

To evaluate the uncertainties associated with the process, a Monte Carlo analysis was performed to compare end point results of the scenarios fossil-PBAT_S and 50%PV + bio-PBAT_S. Pedigree matrixes are reported in Tables S15−S17. Results confirm that fossil-PBAT_S performs better than 50%PV + bio-PBAT_S over 95% of the 1000 runs of the analysis in the Ecosystems category, while for the Resources category fossil-PBAT_S has higher impacts 99% of the time. On the Human health issue, it is not possible to distinguish the impacts associated with the two scenarios, as fossil-PBAT_S had higher impacts than 50%PV + bio-PBAT_S 50% of the time. Results are depicted in Figure 6 and Table S20.

**Figure 6:**
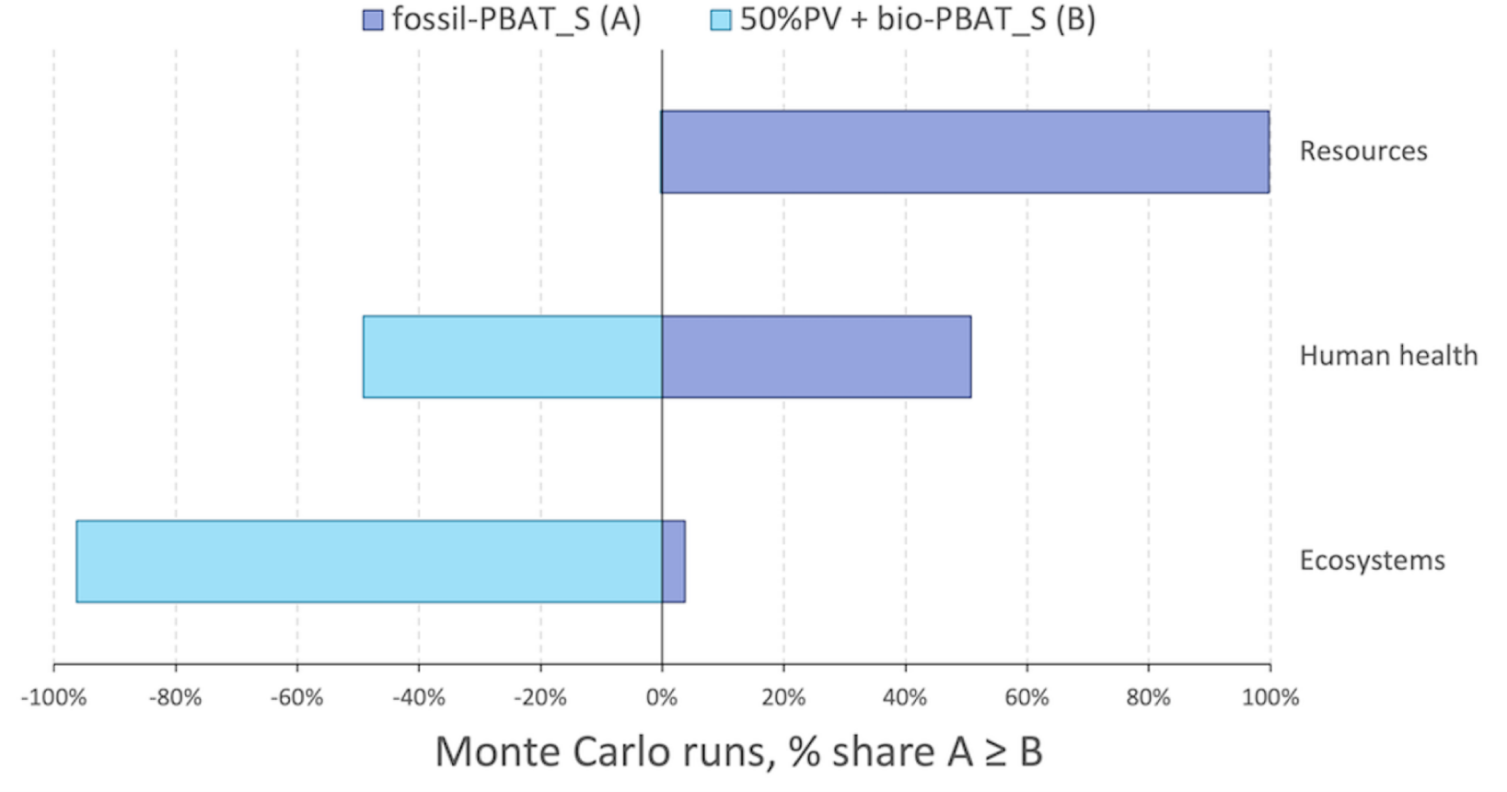
Results of the Monte Carlo analysis conducted to compare the end point environmental impacts of scenario fossil-PBAT_S (A) to scenario 50%PV + bio-PBAT_S (B); method: ReCiPe 2016 end point (H) v1.11/World 2010 (H/A).

The life cycle analysis of a PBAT composite reinforced with inulin allowed to quantify the potential environmental impacts of its production process at laboratory scale and to identify the most relevant environmental hotspots. On average, the upstream production of PBAT resulted into the highest contribution to the overall impacts of the composite. On a process level, potential strategies to reduce the impacts of the composite before an eventual deployment at industrial scale involve the optimization of the twin screw extrusion and PBAT milling stages, after which part of the initial PBAT results unsuitable for the intended application and is discarded as scrap. At the current stage, 1.32 kg of PBAT are required to obtain the 0.95 kg polymeric matrix of the composite, resulting into a 72% efficiency in terms of mass. Increasing the efficiency of the process to integrate 95% of the initial PBAT into the composite would lead to a potential cumulative reduction of the cradle-to-gate carbon footprint of the composite by 24%. Other potential upstream impact reduction routes involve employing PBAT from renewable sources, valorizing the source of inulin in its entirety and increasing the share of renewable electricity consumed in the production process of the composite.

Since the scope of this study is limited to the production process of the PBAT:inulin composite, cradle-to-gate system boundaries were adopted, excluding the use and the end-of-life stages. Further testing on the mechanical properties and degradation pathways of the composite, which will be the objects of future studies, are needed to thoroughly assess the potential applications and waste valorization strategies.

However, in order to inform the readers, the environmental profile of the composite material has been compared to the production of fossil-based LDPE, considered as the incumbent industrial benchmark, to evaluate potential environmental advantages and shortcomings. The discussion is reported in the Supporting Information and depicted in Figure S11, along with additional qualitative considerations on the potential impacts of the end-of-life stage of the composite (section “Potential for scalability and environmental relevance”).

## Conclusions

The purpose of this study was to investigate the mechanical and environmental performance of a biodegradable PBAT-inulin composite material. The production process selected and carried out to make the PBAT-inulin composite films involves laboratory equipment that can easily be scaled up. Grinding, sieving, twin-screw extrusion, and injection molding are all commonly used processes for producing large quantities of polymer materials. To conduct the structural and mechanical analysis, a process to integrate PBAT with inulin D and T types at different concentration levels was proposed, and the mechanical properties of stress and strain at break and Young’s modulus were measured. The correlation between inulin type and concentration and mechanical properties was then evaluated by means of a DoE-based statistical analysis, finding that inulin concentration, rather than inulin type, has the most significant impact on the final properties of the composite, entailing a decrease in strain at break as the amount of inulin increased. From the model results, an optimal amount of inulin equal to 4.4−4.5% is found. To integrate mechanical and structural analysis with an evaluation of the environmental profile, a cradle-to-gate LCA was applied to investigate the manufacturing process of the composite with a 5% inulin content. Results show that fossil-based PBAT production has the highest average impacts (68%) per kg of composite across all evaluated midpoint categories, except for MEP and LOP where biobased inulin production entailed the highest contribution. Furthermore, the evaluation of different scenarios employing biobased PBAT and an increased quota of renewable electricity for the manufacture of the composite shows a reduction in the carbon footprint of the composite, along with potential trade-offs due to the increase in the MEP and LOP categories linked to the consumption of biobased feedstock. Future studies investigating the biodegradability of the PBAT-inulin composite are needed to thoroughly assess all the potential end-of-life strategies. From an LCA perspective, this would also inform quantitative cradle-to-grave studies, allowing comprehensive comparisons on the environmental performance of the composite against fossil-based commercial solutions. Ultimately, the adoption of an integrated approach allows for assessing both the mechanical and environmental properties of the PBAT-inulin composite under study, providing valuable information toward the implementation of optimization strategies for future technological development of the material.

## Supplementary Material



## Notes

Additional context, complete inventories of the modeled processes, and additional results for the life cycle analysis (PDF)
