# Supplementary material for: Structural, Mechanical, and Environmental Assessment of a Poly(butylene adipate-co-terephthalate) (PBAT)-Inulin Composite Material
Source: ACS Sustain Chem Eng. 2026 Mar 11;14(11):5591–600. doi: 10.1021/acssuschemeng.5c13077 (PMC13015832; doi:10.1021/acssuschemeng.5c13077)
Supplement: Supplementary file 1 [file sc5c13077_si_001.pdf]

Supplementary Material: Structural, mechanical and environmental assessment of a poly(butylene adipate-co-terephthalate) (PBAT)-Inulin composite material

Francesco Brenda[a], Silvia Barbi[b], Monia Montorsi[b], Duccio Gallichi Nottiani[c,d], Maria Grimaldi[c,d], Olimpia Pitirollo[e], Corrado Sciancalepore[c,d], Daniel Milanese[c,d,f], Daniele Cespi[a,d,f,]*

a) Department of Industrial Chemistry "Toso Montanari", University of Bologna, Via Piero Gobetti, 85, 40129, Bologna, Italy
b) Department of Science and Methods for Engineering, University of Modena and Reggio Emilia, Via Amendola 2, 42122-Reggio Emilia, Italy
c) Department of Systems Engineering and Industrial Technologies – DISTI, University of Parma, Parco Area delle Scienze 181/A, 43124, Parma, Italy
d) National Interuniversity Consortium of Materials Science and Technology (INSTM), Via G. Giusti 9, 50121, Florence, Italy
e) Department of Chemistry, Life Science, and Environmental Sustainability – SCVSA, University of Parma, Parco Area delle Scienze 17/A, 43124, Parma, Italy
f) Interdepartmental Centre of Industrial Research "Renewable Resources, Environment, Sea and Energy", University of Bologna, Via Dario Campana, 71, 47922, Rimini, RN, Italy

[*]Corresponding authors: daniele.cespi2@unibo.it

Number of pages: 35

Number of figures: 11

Number of tables: 20

Additional context – LCA of PBAT and its monomers

Wang et al. (2024) compared the carbon footprint (CF) of fossil and bio-based PBAT,(1)(1) finding that the percentage contribution of the three monomers varied based on whether they were produced from fossil or biogenic feedstock, i.e. fossil adipic acid was the highest contributor to fossil PBAT CF while bio-terephthalic acid reported the most significant share of impacts in bio-PBAT synthesis. The authors also performed a techno-economic analysis (TEA), finding that 90% of the total production cost of both fossil and bio-based PBAT is associated with the cost of monomers. Ratshoshi et al. (2024) performed a TEA to study the economic feasibility of PBAT produced in an integrated biorefinery able to synthesize all monomers starting from sugar molasses, finding that bio-based PBAT could be potentially cost-competitive with its fossil counterpart. (2)(2) The production of fossil PBAT in China was also investigated via LCA and compared to PBAT produced via second-generation biomass feedstock (Luo et al., 2024); (3)(3) results of the analysis showed that monomers production entailed most environmental impacts across all categories, and that substituting fossil monomers with second generation bio-based alternatives could lead to a reduction in overall impacts.

From an environmental perspective, performances of PBAT-based composites were also assessed in literature via LCA: the use of biochar or carbon black as fillers for PBAT-based mulch films was assessed, both on a technical and environmental basis, finding that the production of the polymer entails the majority of impacts on average across the considered categories on a cradle-to-gate basis (Hernandez-Charpak et al., 2024). (4) The reinforcement of PBAT with inorganic fillers has also been studied from an environmental perspective: Akbarian-Saravi et al. assessed the reinforcement of PBAT with talc, silica and magnesium under a gate-to-gate scope, highlighting the contribution of the filler integration steps to the overall impacts of the composite. The authors also compared landfilling and industrial composting as end-of-life options for the composite, reporting environmenal benefits for composting associated with the avoided production of inorganic fertilizers, (5) Hasan et al. also assessed the production of PBAT based film reinforced with talc, considering cradle-to-grave system boundaries, finding that substituting fossil monomers with bio-based alternatives would entail a reduction in the environmental impacts associated with the composting at end-of-life stage. (6) Zhou et al. analyzed the cradle-to-gate carbon footprint of the production of a ternary PBAT-montmorillonite-lignin film, reporting that the integration of these fillers would reduce the carbon footprint of the composite compared to pure PBAT; however, on a cradle-to-gate basis, the impacts of the composite would still be higher than a conventional

polyethylene film, (7) The use of PBAT has also been investigated from a mechanical and environmental perspective as a biodegradable component of multilayered plastic materials. (8) Table S2 summarizes the methodological assumptions and the carbon footprint results of the reported LCA studies.

In the published literature, the potential environmental impacts associated with the production of bio-based PBAT monomers were investigated by means of LCA. Volanti et al. (2019) reported that the production of bio-terephthalic acid could potentially become competitive from an environmental standpoint if its production was based on waste biomass, (9) while on the other hand adopting feedstock from dedicated cropland could hamper the mitigation efforts compared to the conventional process. Regarding the production of bio-based 1,4-butanediol from wheat straw, (10) the environmental hotspots were found in the cultivation of biobased feedstock and heat requirements of the biorefinery, and, overall, the biobased alternative was found to reduce potential environmental impacts compared to fossil 1,4-butanediol on a cradle-to-gate basis. The production of bio-based adipic acid from the valorization of lignin was also investigated, (11) finding a potential reduction in environmental impacts compared to the fossil route, due to a mitigation in the consumption of fossil fuels and of N_2O emissions resulting from the conventional synthetic pathway.

Table S1: Complete Experimental Plan

Run	Inulin Type	Quantity (%)	Stress at break (MPa)	Strain at break (%)	Young Module (MPa)
1	D	0	9.25	168.27	128.14
2	D	0	9.35	173.77	126.21
3	D	0	9.28	168.68	129.62
4	D	0	9.45	146.26	134.55
5	D	0	8.94	107.77	115.57
6	T	0	9.14	121.67	114.53
7	T	0	9.39	143.84	118.17
8	T	0	9.34	182.36	122.08
9	T	0	9.24	91.33	123.51
10	T	0	9.14	172.01	117.78
11	D	5	8.37	31.84	136.41
12	D	5	8.19	35.06	133.94
13	D	5	8.42	29.53	137.08
14	D	5	8.25	45.41	132.58
15	D	5	8.17	34.89	134.19
16	D	5	8.25	33.36	131.65
17	D	5	8.25	28.17	132.40

18	D	5	8.20	26.76	134.91
19	D	5	8.07	23.68	131.96
20	D	5	8.13	22.87	133.43
21	D	10	7.95	20.14	153.49
22	D	10	7.81	18.45	153.99
23	D	10	7.97	19.83	155.26
24	D	10	7.80	14.67	156.73
25	D	10	7.89	16.64	156.14
26	D	10	7.79	16.16	153.75
27	D	10	7.68	14.70	152.98
28	D	10	8.16	15.89	156.99
29	D	10	7.85	16.11	156.00
30	D	10	7.93	20.34	156.85
31	T	5	8.25	26.10	139.63
32	T	5	8.25	24.07	138.36
33	T	5	8.23	21.79	138.81
34	T	5	8.02	25.47	136.07
35	T	5	8.47	26.37	144.77
36	T	5	8.37	26.975	137.77
37	T	5	8.10	25.71	138.57
38	T	5	8.36	24.95	142.57
39	T	5	8.16	23.99	143.71
40	T	5	8.06	22.16	144.08
41	T	10	7.53	17.35	161.54
42	T	10	7.37	18.66	160.55
43	T	10	7.40	19.69	160.48
44	T	10	7.48	18.62	162.78
45	T	10	7.46	17.28	164.32
46	T	10	7.57	14.63	163.35
47	T	10	7.24	16.39	162.00
48	T	10	7.39	16.19	163.95
49	T	10	7.27	17.18	164.01
50	T	10	7.32	18.93	164.38

Table S2: Summary of methodological choices and calculated carbon footprints of previously published literature on LCA applications to PBAT composites and products.

Authors (Year)	Material	System boundaries	Functional unit	Carbon footprint	LCIA method
**Wang et al. (2024)**	PBAT (fossil or bio-based)	Cradle-to-gate	1 kg of PBAT granulate (process simulation)	8.22 kg CO_2 eq./kg PBAT (fossil); 4.43 kg CO_2 eq./kg PBAT (bio)	ReCiPe 2016
**Luo et al. (2024)**	PBAT (fossil or bio-based)	Cradle-to-gate	1 kg of PBAT granulate produced in China	5.89 kg CO_2 eq./kg PBAT (fossil); 3.72 kg CO_2 eq./kg PBAT (bio)	ReCiPe 2016
**Hernandez-Charpak et al. (2024)**	Biochar reinforced PBAT composite film	Cradle-to-gate	1 ha (6000 m^2) of mulched agricultural land	1617 kg CO_2 eq./ha	ReCiPe 2016
**Zhou et al. (2024)**	Montmorillonite and lignin reinforced PBAT composite film	Cradle-to-gate	1 kg of film	6 kg CO_2 eq./kg film	N/A
**Akbarian-Saravi et al. (2025)**	Talc reinforced PBAT composite resin	Gate-to-gate/grave	1 kg of resin	8.64 kg CO_2 eq./kg resin (gate-to-gate); -2.71 kg CO_2 eq. Including EoL composting	ReCiPe 2016, IPCC 2021
**Hasan et al. (2025)**	Talc reinforced PBAT composite film	Cradle-to-grave	1 kg of PBAT-talc film	22.1 kg CO_2 eq./kg film (including (EoL)	TRACI 2.1
**Abdelwahab et al. (2025)**	Multilayered plastics containing PBAT	Cradle-to-gate	1 m^2 of produced film	0.2-0.6 kg CO_2 eq./m^2 film (several compositions tested)	TRACI 2.1

Supplementary materials and methods, LCA: life cycle inventories (LCI) modelling data

Life cycle inventory for the production of the composite was modelled based on production data obtained in the laboratory relative to the material and energy flows. For the electricity consumption, the Italian 2023 electricity mix (IEA, 2023) was employed as the manufacture of the composite takes place in UniPr laboratories. (12) Complete inventory, considering all composite manufacturing process steps, is reported in Table S3.

Table S3: Inventory relative to the production of 1 kg of PBAT:inulin (95:5) composite.

Processing phase	Input	Amount	Unit	Process
PBAT DRYING	PBAT	1.3E+00	kg	Modified PET process
	Electricity	1.8E+02	Wh	Italy, IEA 2023
PBAT MILLING	Electricity	3.3E+02	Wh	Italy, IEA 2023
	Nitrogen, inert gas	8.4E-01	kg	
INULIN DRYING	Inulin	8.0E-02	kg	Hinghsamer et al., 2022
	Electricity	1.8E+02	Wh	Italy, IEA 2023
INULIN SIEVING	Electricity	1.3E+02	Wh	Italy, IEA 2023
COMPOSITE DRYING	Electricity	3.5E+02	Wh	Italy, IEA 2023
COMPOSITE TWIN SCREW EXTRUSION + AIR COOLING + WINDING DRUM	Electricity	3.9E+03	Wh	Italy, IEA 2023
COMPOSITE SHREDDING	Electricity	1.9E+02	Wh	Italy, IEA 2023
Processing phase	**Output**	**Amount**	**Unit**	**Process**
Overall	PBAT:Inulin 95:5 composite	1.0E+00	kg	Product
PBAT DRYING	H_2O, in air	1.0E-02	kg	Emissions to air
PBAT MILLING	N_2, in air	8.4E-01	kg	Emissions to air
	PBAT scrap	6.0E-02	kg	Hazardous waste, for incineration {Europe without Switzerland}| treatment of hazardous waste, hazardous waste incineration | APOS, U
INULIN DRYING	H_2O, in air	3.9E-03	kg	Emissions to air
INULIN SIEVING	Inulin scrap	1.0E-02	kg	Hazardous waste, for incineration {Europe without Switzerland}| treatment of hazardous waste, hazardous waste incineration | APOS, U
COMPOSITE DRYING	H_2O, in air	7.0E-02	kg	Emissions to air
COMPOSITE TWIN SCREW EXTRUSION + AIR COOLING + WINDING DRUM	Composite scrap	2.6E-01	kg	Hazardous waste, for incineration {Europe without Switzerland}| treatment of hazardous waste, hazardous waste incineration | APOS, U
COMPOSITE SHREDDING	Composite scrap	5.0E-02	kg	Hazardous waste, for incineration {Europe without Switzerland}| treatment of hazardous waste, hazardous waste incineration | APOS, U

The production of PBAT was modelled by modifying the ecoinvent process for the production of PET, assuming similar energy and auxiliary requirements for the polymerization process, as reported in the published literature. (4)(4) Terephthalic acid (TA) inputs were substituted with adipic acid (AA) and 1,4-butanediol (1,4-BDO), following the ratio reported in Schrijvers et al. (13)The energy and auxiliary requirements for the polymerization process of PET in ecoinvent were maintained equal. Streamlined inventory is reported in Table S4.

Table S4: Inventory relative to the production of 1 kg of PBAT.

**Input**	**Amount**	**Unit**	**Process**	**Database**
Adipic acid	0.37	kg	AA, fossil or bio	ecoinvent v3.10
Terephtalic acid	0.33	kg	TA, fossil or bio	ecoinvent v3.10
1,4-butanediol	0.41	kg	1,4-BDO, fossil or bio	ecoinvent v3.10
**Output**	**Amount**	**Unit**	**Process**	**Database**
PBAT	1	kg	PBAT production	

The production of inulin was modelled according to the life cycle analysis performed by Hingshamer et al., (14) based on the extraction process starting from chicory roots, to model the energy requirements and material consumption associated with the process. Given that the production of chicory takes place in the Netherlands, background processes representative of the Netherlands and the European region were selected when possible. Full inventory is reported in Table S5.

Table S5: Inventory relative to the production of 1 ton of inulin.

Input	Amount	Unit	Process	Database
Chicory roots	6.30E+00	ton	Chicory roots, at farm {NL} Economic, U	Agri-footprint
Active carbon	8.50E-04	ton	Activated carbon, granular {GLO}| market for activated carbon, granular | APOS, U	ecoinvent v3.10
Flocculant	4.70E-04	ton	Non-ionic surfactant {GLO}| market for non-ionic surfactant | APOS, U	ecoinvent v3.10
NaOH	1.20E-01	ton	Sodium hydroxide, without water, in 50% solution state {RER}| market for sodium hydroxide, without water, in 50% solution state | APOS, U	ecoinvent v3.10
HCl	9.80E-02	ton	Hydrochloric acid, without water, in 30% solution state {RER}| market for hydrochloric acid, without water, in 30% solution state | APOS, U	ecoinvent v3.10
Natural gas	3.00E+01	Nm^3	Natural gas, high pressure {NL}| market for natural gas, high pressure | APOS, U	ecoinvent v3.10
Electricity	2.00E+00	MWh	Electricity, medium voltage {NL}| market for electricity, medium voltage | APOS, U	ecoinvent v3.10
**Output**	**Amount**	**Unit**	**Process**	**Database**
Inulin	1.0E+00	ton	_Inulin production, extraction from chicory roots	
Wastewater to treatment	4.6E+00	m^3	Wastewater, average {Europe without Switzerland}| market for wastewater, average | APOS, U	ecoinvent v3.10
Biomass waste	6.8E-01	ton	Biowaste {RoW}| market for biowaste | APOS, U	ecoinvent v3.10
CO_2	6.0E-02	ton	Carbon dioxide, fossil, to air	
Other	4.7E-04	ton	Organic substances, unspecified, to water	
Sodium (I)	6.9E-01	ton		
Chloride	9.5E-02	ton		

The TA via bio-based route was modelled starting from the inventory reported in Volanti et al., (9)who simulated the production of bio-TA starting from sweet corn as bio-based feedstock. The inventory simulated the fermentation of corn to produce iso-butanol, followed by its conversion into para-xylene via Gevo® process and subsequent production of TA via Amoco® process. Complete inventory is reported in Table S6.

Table S6: Inventory relative to the production of 1 kg of bio-TA.

Input	Amount	Unit	Process	Database
Corn	8.9E+02	kg	Sweet corn {RoW}| market for sweet corn | APOS, U	ecoinvent v3.10
Water	1.2E+03	kg	Water, process, unspecified natural origin/kg	
Carbon dioxide, in air	3.5E-01	kg	Carbon dioxide, in air	
NaOH	9.9E+00	kg	Sodium hydroxide, without water, in 50% solution state {RER}| market for sodium hydroxide, without water, in 50% solution state | APOS, U	ecoinvent v3.10
H_2SO_4			Sulfuric acid {RER}| market for sulfuric acid | APOS, U	ecoinvent v3.10
Ammonium sulfate	2.6E+00	kg	Ammonium sulfate {RER}| market for ammonium sulfate | APOS, U	ecoinvent v3.10
Diammonium phosphate	2.6E+00	kg	Diammonium phosphate {RER}| market for diammonium phosphate | APOS, U	ecoinvent v3.10
Acetic acid	5.4E-01	kg	Acetic acid, without water, in 98% solution state {GLO}| market for acetic acid, without water, in 98% solution state | APOS, U	ecoinvent v3.10
Oxygen	5.9E+01	kg	Oxygen, liquid {RER}| market for oxygen, liquid | APOS, U	ecoinvent v3.10
Electricity, high voltage	9.5E+01	kWh	Electricity, high voltage {RER}| market group for electricity, high voltage | APOS, U	ecoinvent v3.10
Heat	2.8E+03	MJ	Heat, district or industrial, natural gas {Europe without Switzerland}| heat production, natural gas, at industrial furnace low-NOx >100kW | APOS, U	ecoinvent v3.10
Cooling energy	8.1E+02	MJ	Cooling energy {CH}| cooling energy, from natural gas, at cogen unit with absorption chiller 100kW | APOS, U	ecoinvent v3.10
**Output**	**Amount**	**Unit**	**Process**	**Database**
TA	1.0E+03	kg		
Carbon dioxide, biogenic	6.1E+02	kg	Emission to air	
Heat, waste	9.3E+02	MJ	Emission to air	
Oxygen	1.5E+00	kg	Emission to air	
Sewage to WWT	4.4E-01	m^3	Wastewater from maize starch production {RoW}| market for wastewater from maize starch production | APOS, U	ecoinvent v3.10
Wastewater average	7.60E-02	m^3	Wastewater, average {Europe without Switzerland}| market for wastewater, average | APOS, U	ecoinvent v3.10

The production of bio-1,4-butanediol (1,4-BDO) was modelled from the inventory reported in Forte et al., (10) who conducted an LCA to evaluate the environmental impacts of the production of 1,4-BDO from wheat straw in Italy. Complete LCI is reported in Table S7.

Table S7: Inventory relative to the production of 1 kg of bio-1,4-BDO.

Input	Amount	Unit	Process	Database	Notes
wheat straw	4.5E+00	kg	Wheat straw, at farm {IT} Economic, U	Agri-footprint	Assuming 44% carbon content in wheat straw and 2 kg of biogenic carbon in WS feedstock
Water	5.8E+00	kg	Water, process, unspecified natural origin/kg		
Sulphuric acid	6.0E-02	kg	Sulfuric acid {RER}| market for sulfuric acid | APOS, U	ecoinvent 3.10	
Nutrients, organic chemicals	3.0E-01	kg	Chemical, organic {GLO}| market for chemical, organic | APOS, U	ecoinvent 3.10	
Heat	1.7E+01	MJ	Heat, district or industrial, natural gas {RER}| market group for heat, district or industrial, natural gas | APOS, U	ecoinvent 3.10	
Lignin residue incineration	2.4E+01	MJ	_Heat, district or industrial, other than natural gas EMISSIONS, WASTE, INFRASTRUCTURE {IT}| heat and power co-generation, wood chips, 6667 kW, state-of-the-art 2014 | APOS, U	ecoinvent 3.10	Emissions only
**Output**	**Amount**	**Unit**	**Process**	**Database**	**Notes**
1,4 BDO	1.0E+00	kg	Product		80% conversion from cellulose and hemicellulose
Wastewater	5.8E+00	L	Wastewater, average {Europe without Switzerland}| market for wastewater, average | APOS, U	ecoinvent 3.10	Completion of mass balance

The production of bio-based adipic acid (AA) was modelled based on the work of Unlu et al., (15) who simulated the production of AA starting from glucose as a bio-based feedstock. The input flows for the hydrogenation catalyst were taken from Corona et al., (11) and Vardon et al. (16) The inventory is reported in Table S8.

Table S8: Inventory relative to the production of 1 kg of bio-AA.

Input	Amount	Unit	Process	Database	Notes
Glucose	1.2E+00	kg	Glucose {GLO}| market for glucose | APOS, U		Input
Water, make-up	1.5E-01	kg	Tap water {Europe without Switzerland}| market for tap water | APOS, U		Make up
Acetic acid	6.7E-02	kg	Acetic acid, without water, in 98% solution state {GLO}| market for acetic acid, without water, in 98% solution state | APOS, U		Make up, solvent
H_2	5.6E-02	kg	Hydrogen, gaseous, medium pressure, merchant {RER}| market for hydrogen, gaseous, medium pressure, merchant | APOS, U		Make up
Rh/C	1.1E-07	kg	Rhodium {GLO}| market for rhodium | APOS, U		Corona et al., hydrogenation catalyst flow, 1% Rh load (Vardon et al)
Active carbon	1.1E-05	kg	Activated carbon, granular {GLO}| market for activated carbon, granular | APOS, U		Vardon et al. https://doi.org/10.1039/C5GC02844B
Heating, natural gas	1.0E+00	MJ	Heat, district or industrial, natural gas {Europe without Switzerland}| heat and power co-generation, natural gas, 1MW electrical, lean burn | APOS, U		
Cooling, natural gas	9.5E+00	MJ	Cooling energy {RoW}| cooling energy, from natural gas, at cogen unit with absorption chiller 100kW | APOS, U		Energy consumption assumed to be coming from natural gas
Electricity, natural gas	9.4E-01	MJ	Cooling energy {RoW}| cooling energy, from natural gas, at cogen unit with absorption chiller 100kW | APOS, U		
**Output**	**Amount**	**Unit**	**Process**	**Database**	**Notes**
Adipic Acid	1.0E+00	kg			Product
WWT	9.7E-01	kg	Cooling energy {RoW}| cooling energy, from natural gas, at cogen unit with absorption chiller 100kW | APOS, U		From reaction+waste

Considerations on the mechanical properties of the composite

Regarding the results of the mechanical properties of the composite material, the incorporation of inulin, a natural polysaccharide, into the poly(butyleneadipate-co-terephthalate) (PBAT) matrix acts as a strategic structural reinforcement, effectively addressing the inherent low stiffness of the neat polyester. While neat PBAT is characterized by high ductility and a low Young's modulus (20-80 MPa), the addition of inulin as a bio-filler has shown to induce a significant enhancement in mechanical properties. The addition of 10% by weight (wt.%) of inulin can lead to an increase in the elastic modulus of over 30% compared to the virgin matrix, depending on the degree of dispersion and the interfacial affinity achieved. This reinforcement effect is attributed to the restricted mobility of the PBAT macromolecular chains, induced by the presence of the rigid inulin particles, as structural anchoring points. When compared to fossil-based benchmarks such as polyethylene (PE), the PBAT/inulin composite exhibits a distinct performance-property trade-off. PE remains superior in terms of absolute mechanical strength ($0.2<E<1.5$ GPa) and thermal stability. However, the developed bio-composite shows stiffness values suitable for specific applications, such as flexible packaging or agricultural mulching films. The mechanical gap between the two materials is compensated by the compostability of the composite material and by the incorporation of inulin, which is based on natural resources.

Supplementary results, DoE

Table S9 – ANOVA

Response	F-test p-value	LoF p-value	Adj-R^2	Pred-R^2	Equation
Stress at break	<0.0001		0.96	0.95	+9.2500 - 0.1371 * Inulin Quantity (D Inulin)<br>+9.2500 - 0.1847 * Inulin Quantity (T Inulin)
Strain at break	<0.0001		0.90	0.88	+ 147.6005 - 13.0203 * Inulin Quantity

Young Module	<0.0001		0.96	0.95	+126.8180 + 2.8400 * Inulin Quantity (D Inulin)<br>+119.2140 +4.3522 * Inulin Quantity (T Inulin)

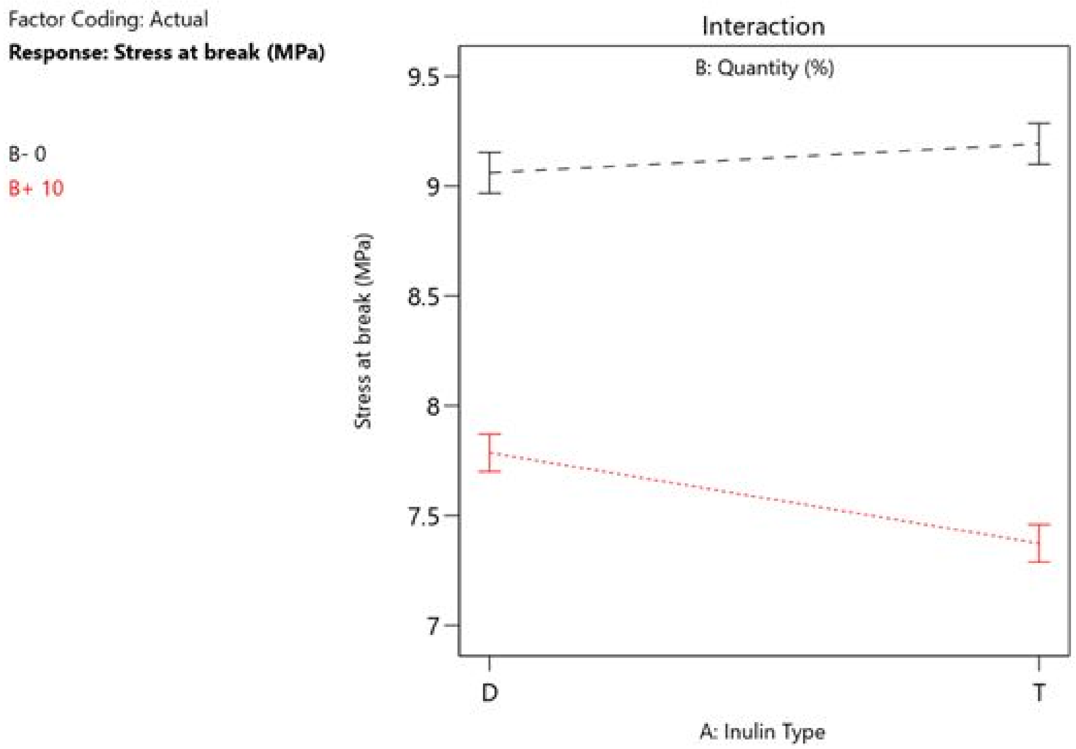

Figure S1 – Interaction plot for Stress at break.

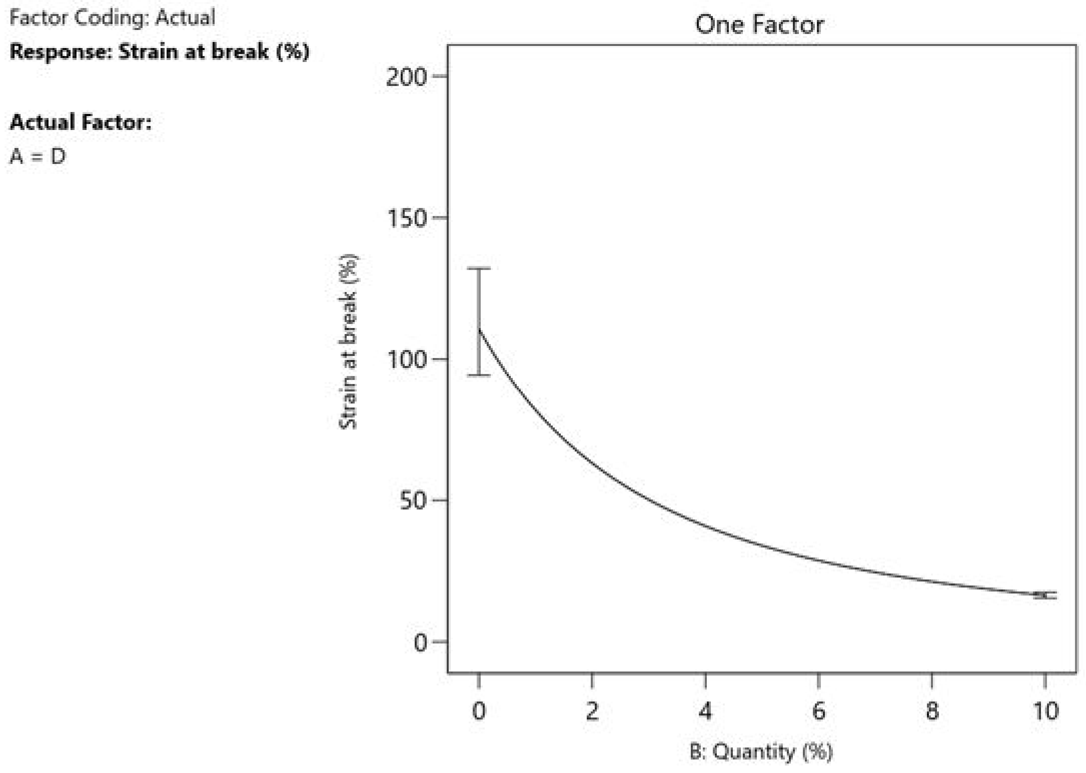

Figure S2 – Interaction plot for Strain at break.

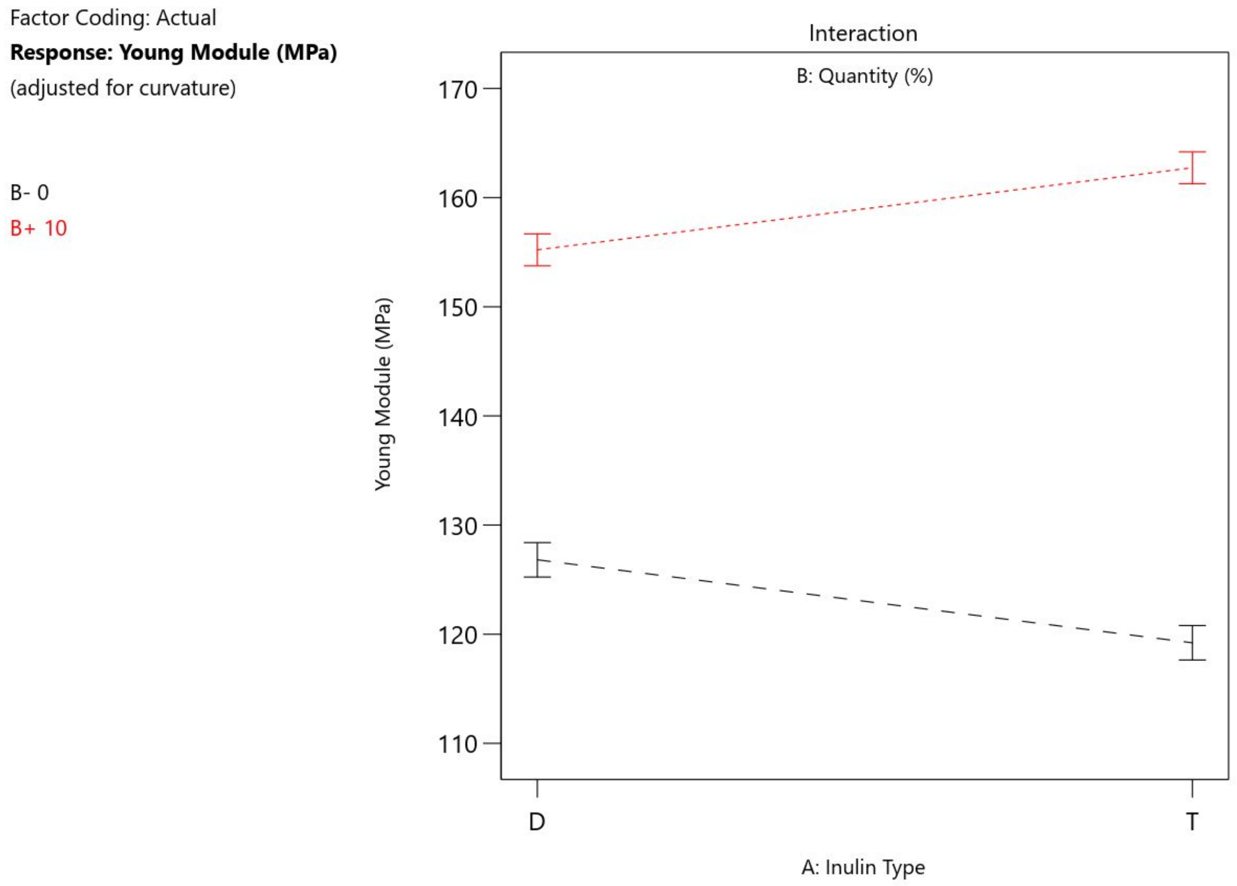

Figure S3 – Interaction plot for Young Module.

LCIA – ReCiPe 2016 results

Midpoint impacts for the considered scenarios are reported in Table S10.

Table S10: PBAT:inulin 95:5 production, midpoint impacts scenarios fossil-PBAT_S, 50%PV_S, bio-PBAT_S, 50%PV + bio-PBAT_S; method: ReCiPe 2016 midpoint (H) v1.11/ World 2010 (H).

Impact category	Unit	fossil-PBAT_S	50%PV_S	bio-PBAT_S	50%PV + bio-PBAT_S
GWP	kg CO_2 eq	1.3E+01	1.2E+01	1.0E+01	9.6E+00
ODP	kg CFC11 eq	8.8E-05	8.8E-05	2.9E-05	2.8E-05
IRP	kBq Co-60 eq	8.1E-01	6.7E-01	8.1E-01	6.7E-01
HOFP	kg NOx eq	1.9E-02	1.8E-02	2.1E-02	2.0E-02
PMFP	kg PM2.5 eq	1.3E-02	1.3E-02	1.5E-02	1.5E-02
EOFP	kg NOx eq	2.1E-02	2.0E-02	2.4E-02	2.3E-02
TAP	kg SO_2 eq	3.5E-02	3.4E-02	6.2E-02	6.0E-02
FEP	kg P eq	3.4E-03	3.3E-03	3.0E-03	2.9E-03
MEP	kg N eq	1.0E-03	9.9E-04	7.7E-03	7.6E-03
TETP	kg 1,4-DCB	1.7E+02	1.7E+02	8.7E+01	8.9E+01
FETP	kg 1,4-DCB	4.8E-01	5.1E-01	5.1E-01	5.4E-01
METP	kg 1,4-DCB	6.7E-01	7.1E-01	6.0E-01	6.4E-01
HTPc	kg 1,4-DCB	1.6E+00	1.5E+00	1.3E+00	1.3E+00
HTPnc	kg 1,4-DCB	1.3E+01	1.3E+01	1.3E+01	1.3E+01
LOP	m^2a crop eq	6.8E-01	6.1E-01	4.4E+00	4.4E+00
SOP	kg Cu eq	2.8E-02	3.0E-02	2.8E-02	3.0E-02
FFP	kg oil eq	3.7E+00	3.4E+00	2.7E+00	2.5E+00
WCP	m^3	2.3E-01	2.1E-01	3.4E-01	3.2E-01

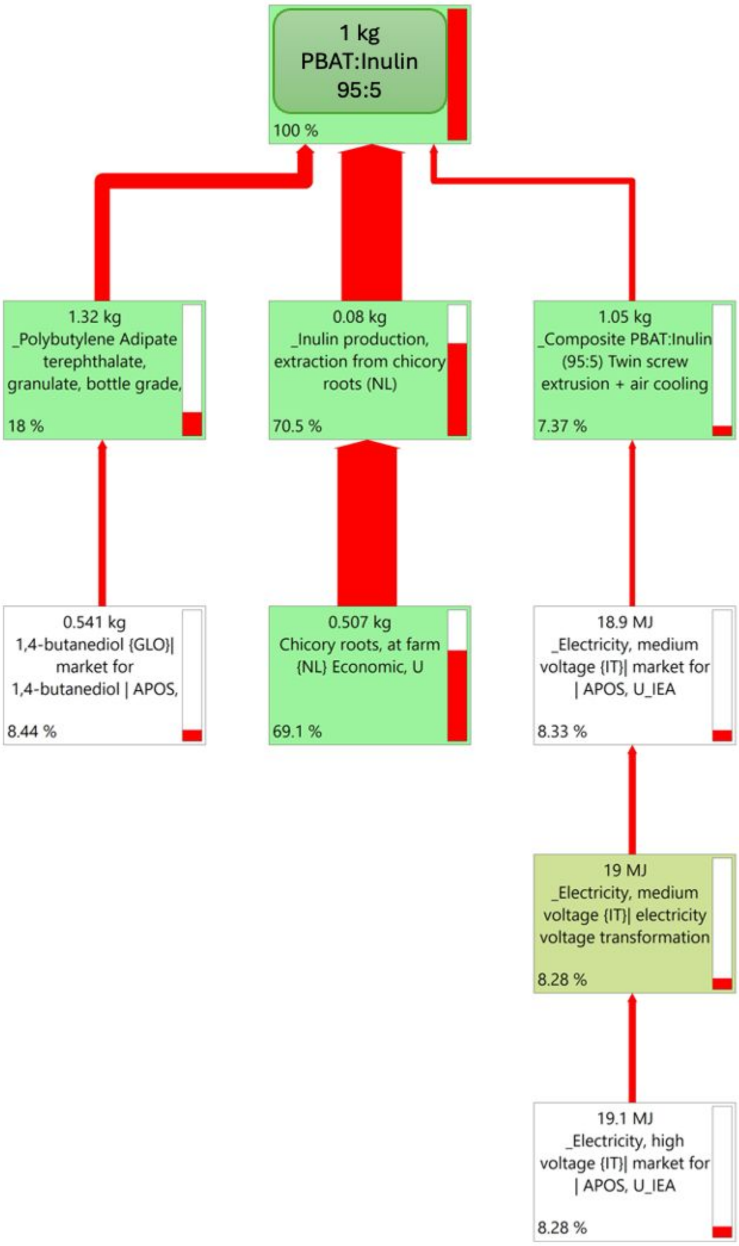

Figure S4: Contribution analysis for 1 kg of composite: MEP category; method: ReCiPe 2016 midpoint (H) v1.11/ World 2010 (H).

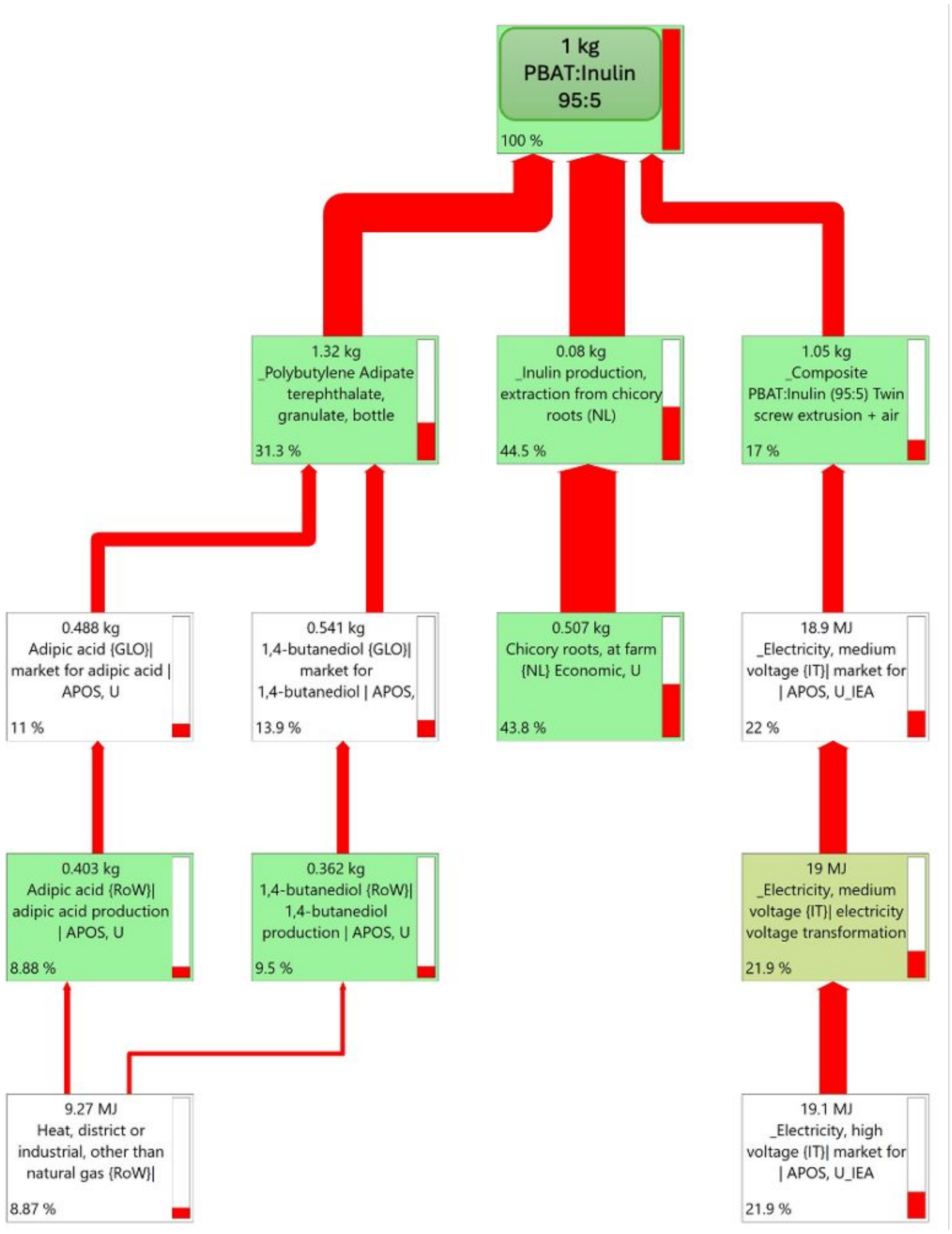

Figure S5: Contribution analysis for 1 kg of composite: LOP category; method: ReCiPe 2016 midpoint (H) v1.11/ World 2010 (H).

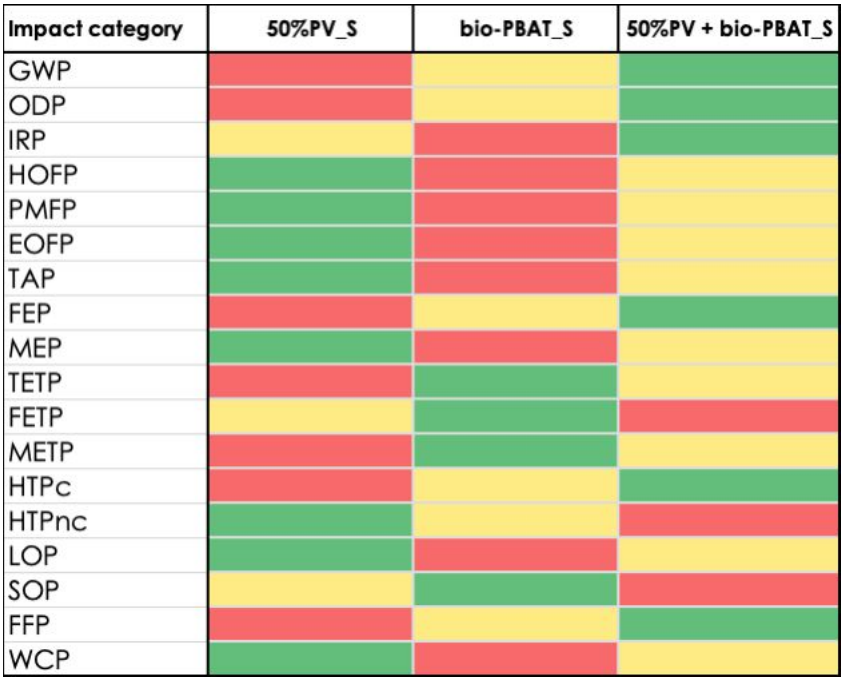

Figure S6: PBAT:Inulin 95:5 production, percentage midpoint impacts of 50%PV_S, bio-PBAT_S and 50%PV + bio-PBAT_S compared to fossil-PBAT_S, FU = 1 kg of composite; method: ReCiPe 2016 midpoint (H) v1.11/ World 2010 (H).

Endpoint impacts for the considered scenarios are reported in Table S11.

Table S11: PBAT:inulin 95:5 production, endpoint impacts of scenarios fossil-PBAT_S, 50%PV_S, bio-PBAT_S, 50%PV+ bio-PBAT_S; method: ReCiPe 2016 endpoint (H) v1.11/ World 2010 (H).

Damage category	Unit	fossil-PBAT_S	50%PV_S	bio-PBAT_S	50%PV + bio-PBAT_S
Human health	DALY	2.9E-05	2.8E-05	2.8E-05	2.7E-05
Ecosystems	species.yr	6.0E-08	5.7E-08	9.1E-08	8.8E-08
Resources	USD2013	1.2E+00	1.1E+00	9.1E-01	8.3E-01

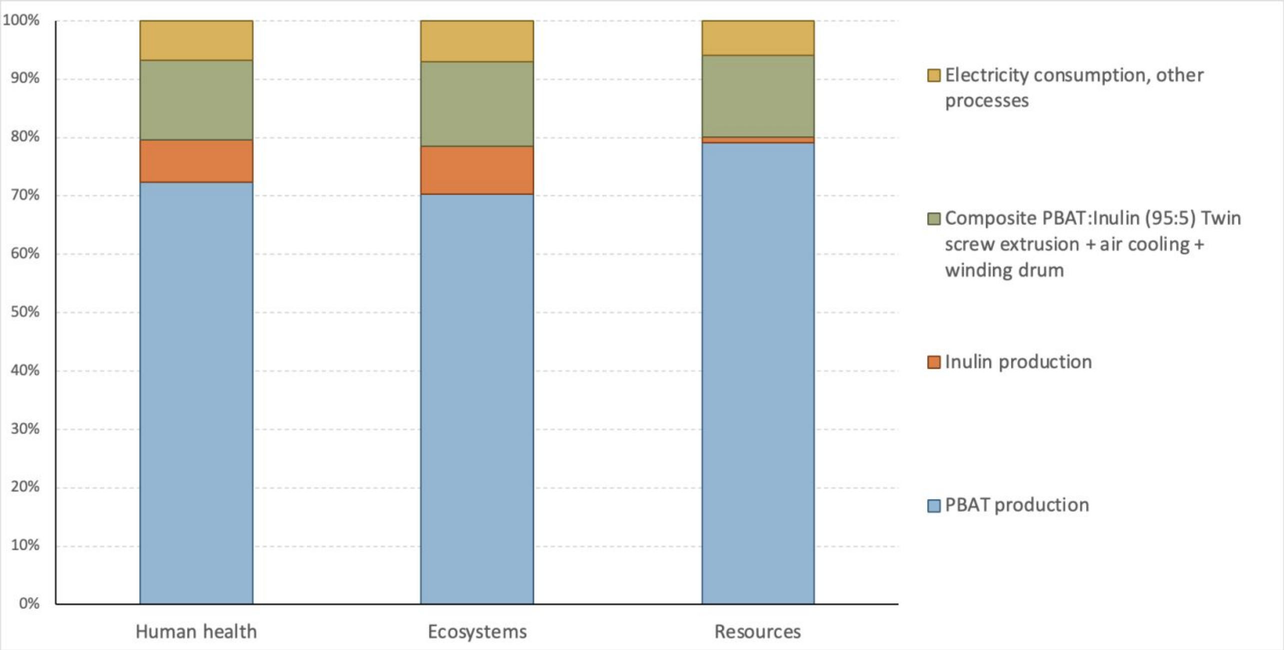

Figure S7: PBAT:inulin 95:5 production, endpoint impacts of scenario fossil-PBAT_S, contribution analysis;method: ReCiPe 2016 endpoint (H) v1.11/ World 2010 (H/A).

Midpoint and endpoint impacts for the 10%Inulin_S compared to fossil-PBAT_S are reported in Figure S8 and Tables S10 and S11, respectively.

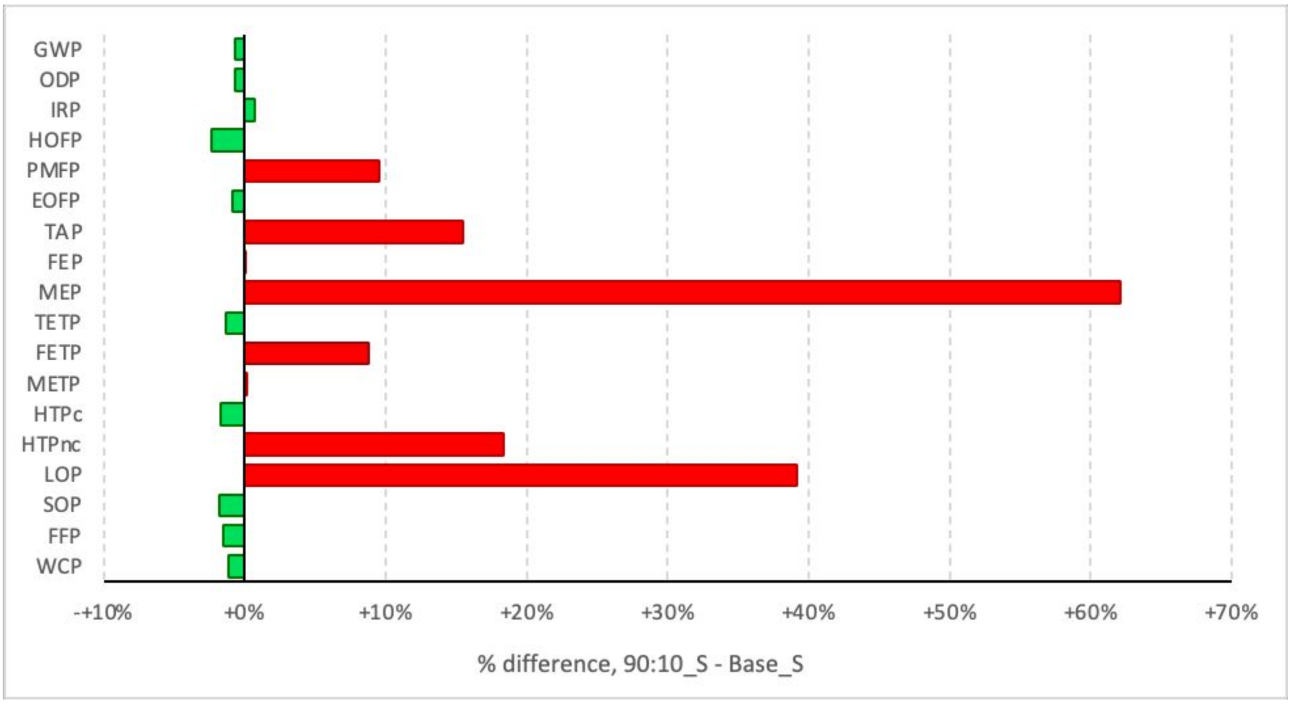

Figure S8: PBAT:Inulin 95:5 and PBAT:Inulin 90:10 production; percentage difference in environmental impact values between 10%Inulin_S and fossil-PBAT_S, evaluated at the midpoint level; method: ReCiPe 2016 midpoint (H) v1.11/ World 2010 (H/A).

Table S12: PBAT:inulin 95:5 production vs PBAT:inulin 90:10, middpoint impacts of scenarios fossil-PBAT_S and 10%Inulin_S; method: ReCiPe 2016 midpoint (H) v1.11/ World 2010 (H).

Impact category	Unit	fossil-PBAT_S	10%Inulin_S	Difference %
GWP	kg CO_2 eq	1.3E+01	1.3E+01	-1%
ODP	kg CFC11 eq	8.8E-05	8.8E-05	-1%
IRP	kBq Co-60 eq	8.1E-01	8.2E-01	1%
HOFP	kg NOx eq	1.9E-02	1.9E-02	-2%
PMFP	kg PM2.5 eq	1.3E-02	1.5E-02	10%
EOFP	kg NOx eq	2.1E-02	2.1E-02	-1%
TAP	kg SO_2 eq	3.5E-02	4.0E-02	15%
FEP	kg P eq	3.4E-03	3.4E-03	0%
MEP	kg N eq	1.0E-03	1.7E-03	62%
TETP	kg 1,4-DCB	1.7E+02	1.7E+02	-1%
FETP	kg 1,4-DCB	4.8E-01	5.2E-01	9%
METP	kg 1,4-DCB	6.7E-01	6.8E-01	0%
HTPc	kg 1,4-DCB	1.6E+00	1.5E+00	-2%
HTPnc	kg 1,4-DCB	1.3E+01	1.5E+01	18%
LOP	m^2a crop eq	6.8E-01	9.4E-01	39%
SOP	kg Cu eq	2.8E-02	2.8E-02	-2%
FFP	kg oil eq	3.7E+00	3.6E+00	-2%
WCP	m^3	2.3E-01	2.3E-01	-1%

Table S13: PBAT:inulin 95:5 production vs PBAT:inulin 90:10, midpoint impacts of scenarios fossil-PBAT_S and 10%Inulin_S; method: ReCiPe 2016 endpoint (H) v1.11/ World 2010 (H).

Damage category	Unit	fossil-PBAT_S	10%Inulin_S	% difference
Human health	DALY	2.9E-05	3.1E-05	5%
Ecosystems	species.yr	6.0E-08	6.3E-08	5%
Resources	USD2013	1.2E+00	1.2E+00	-1%

LCIA – IPCC 2021results

Full results of the GWP impacts of the composite production, calculated with the IPCC 2021 method (including carbon uptake), are reported in Table S14 and Figure S9.

Table S14: Carbon footprint associated with the production of the composite; method: IPCC 2021, GWP100 incl. CO_2 uptake, v.1.01.

Impact category	Unit	Total	PBAT production	Composite PBAT:Inulin (95:5) Twin screw extrusion + air cooling + winding drum	Others
GWP100 - fossil	kg CO_2-eq	1.3E+01	9.6E+00	2.0E+00	1.1E+00
GWP100 - biogenic	kg CO_2-eq	8.7E-01	4.2E-01	2.7E-01	1.7E-01
GWP100 - land transformation	kg CO_2-eq	9.7E-03	5.0E-03	2.9E-03	1.8E-03
GWP100 - CO2 uptake	kg CO_2-eq	-3.6E-01	-2.4E-01	-7.3E-02	-4.7E-02
Total	kg CO_2-eq	1.3E+01	9.7E+00	2.2E+00	1.3E+00

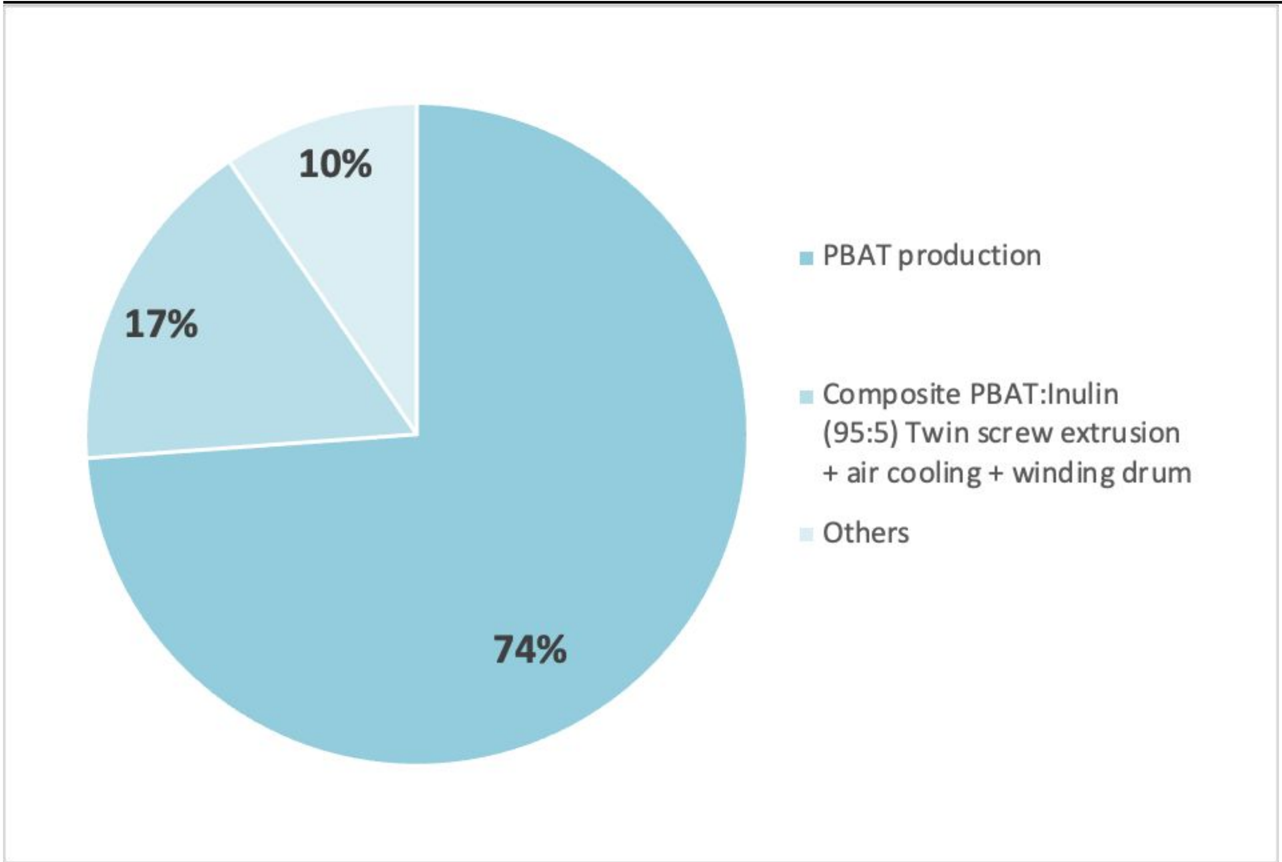

Figure S9: Carbon footprint associated with the production of the composite; method: IPCC 2021, GWP100 incl. CO_2 uptake, v.1.01.

To assess the contribution of polymer precursors to its CF with respect to the FU and the effect of modelling choices on environmental impacts, a focus analysis on the manufacture of PBAT and its monomers was performed to compare the production of fossil-based and bio-based PBAT. The

adoption of fossil-based precursors leads to a CF of 9.7 kg CO_2 eq./FU, of which the production of fossil adipic acid constitutes 48% of the overall carbon emissions, followed by 1,4-butanediol at 36% and terephthalic acid at 12%; the remaining emissions are associated with the utilities employed in the polymerization process. For bio-based PBAT, bio-terephthalic acid from iso-butanol entailed 49% of the polymer CF, followed by bio-1,4-butanediol at 31% and bio-adipic acid at 14%. Results are summarized in Tables S12, S13, and Figure S10. In this study, both TA and AA are assumed to be derived from dedicated crops, i.e. corn, while 1,4-BDO is considered to be produced starting from wheat straw: TA and AA fossil and biogenic CO_2 emissions are mitigated by CO_2 uptake deriving from corn cultivation, while the uptake associated with the cultivation of wheat are limited from the exclusion of carbon storage in crops and from the economic allocation performed on wheat straw inputs. (17)

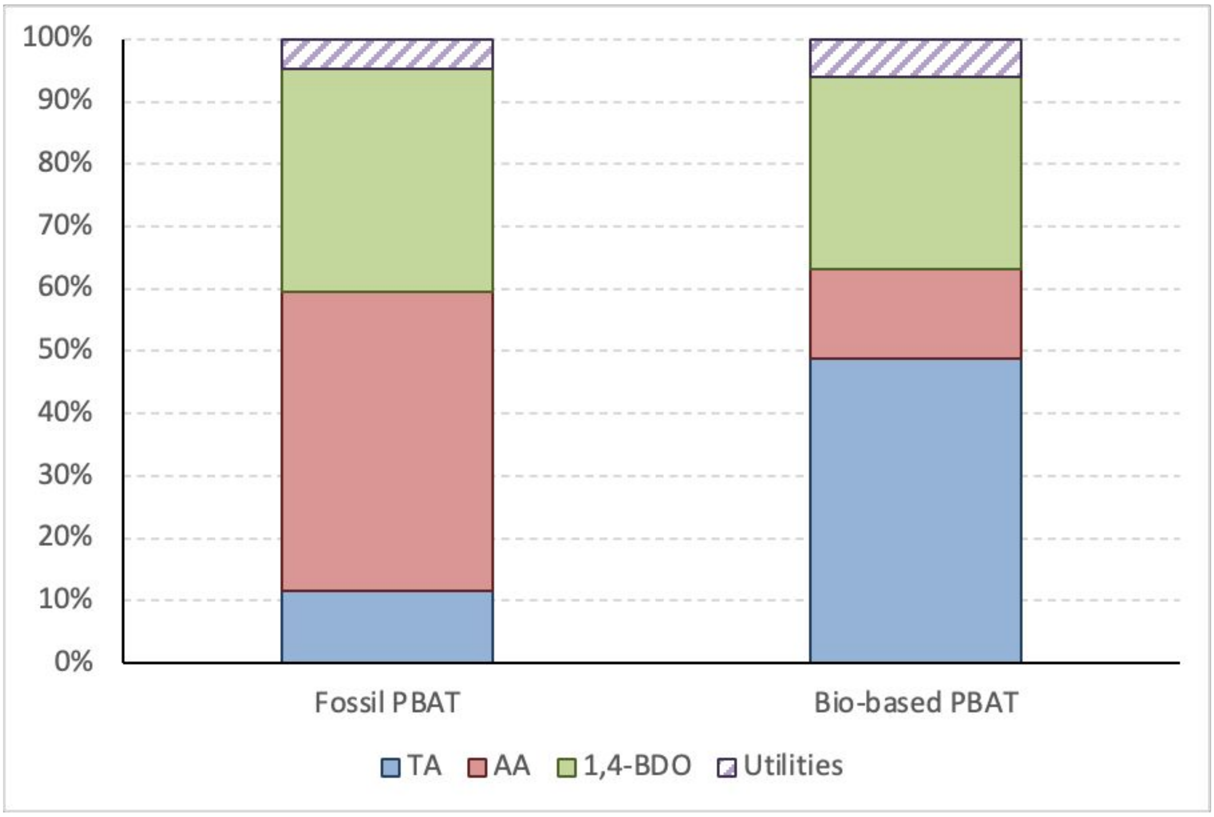

Figure S10: PBAT production, carbon footprint of fossil-based and bio-based processes, contribution analysis of monomers and utilities; method: IPCC 2021, GWP100 incl. CO_2 uptake, v.1.01.

Table S15: Carbon footprint associated with the production of fossil PBAT; method: IPCC 2021, GWP100 incl. CO2 uptake, v.1.01.

Impact category	Unit	Total	Terephtalic acid, fossil	Adipic acid, fossil	1,4-butanediol, fossil	Utilities and process emissions
GWP100 - fossil	kg CO_2-eq	9.6E+00	1.1E+00	4.6E+00	3.4E+00	4.5E-01
GWP100 - biogenic	kg CO_2-eq	4.2E-01	1.7E-02	1.7E-01	2.0E-01	3.1E-02
GWP100 - land transformation	kg CO_2-eq	5.0E-03	6.6E-04	8.6E-04	2.8E-03	5.9E-04
GWP100 - CO_2 uptake	kg CO_2-eq	-2.4E-01	-1.6E-02	-9.0E-02	-1.1E-01	-2.0E-02
TOTAL	kg CO_2-eq	9.7E+00	1.1E+00	4.7E+00	3.5E+00	4.6E-01
	Percentage	100%	12%	48%	36%	5%

Table S16: Carbon footprint associated with the production of bio-based PBAT; method: IPCC 2021, GWP100 incl. CO_2 uptake, v.1.01.

Impact category	Unit	Total	Terephtalic acid, bio, from corn	Adipic acid, bio, from glucose	1,4-butanediol, bio, from wheat straw	Utilities and process emissions
GWP100 - fossil	kg CO_2-eq	6.1E+00	2.5E+00	1.7E+00	1.5E+00	4.5E-01
GWP100 - biogenic	kg CO_2-eq	3.8E+00	2.8E+00	3.0E-02	9.5E-01	3.1E-02
GWP100 - land transformation	kg CO_2-eq	8.1E-01	3.4E-01	4.7E-01	4.0E-04	5.9E-04
GWP100 - CO_2 uptake	kg CO_2-eq	-3.0E+00	-1.9E+00	-1.1E+00	-9.7E-03	-2.0E-02
TOTAL	kg CO_2-eq	7.7E+00	3.8E+00	1.1E+00	2.4E+00	4.6E-01
	Percentage	100%	49%	14%	31%	6%

Potential for scalability and environmental relevance

To extend the discussion on the environmental relevance of the composite compared to commercially available alternatives, the cradle-to-gate impacts of the process were compared to the production of 1 kg of granulate LDPE produced in Europe. For the comparison, the ecoinvent process "Polyethylene, low density, granulate {RER}| polyethylene production, low density, granulate | APOS, U" was selected, to consider an average European production of low-density polyethylene. Comparative results for the ReCiPe 2016 midpoint categories are reported in Figure

S11. For the GWP category, impacts for LDPE are still 80% lower compared to those of the analyzed composite.

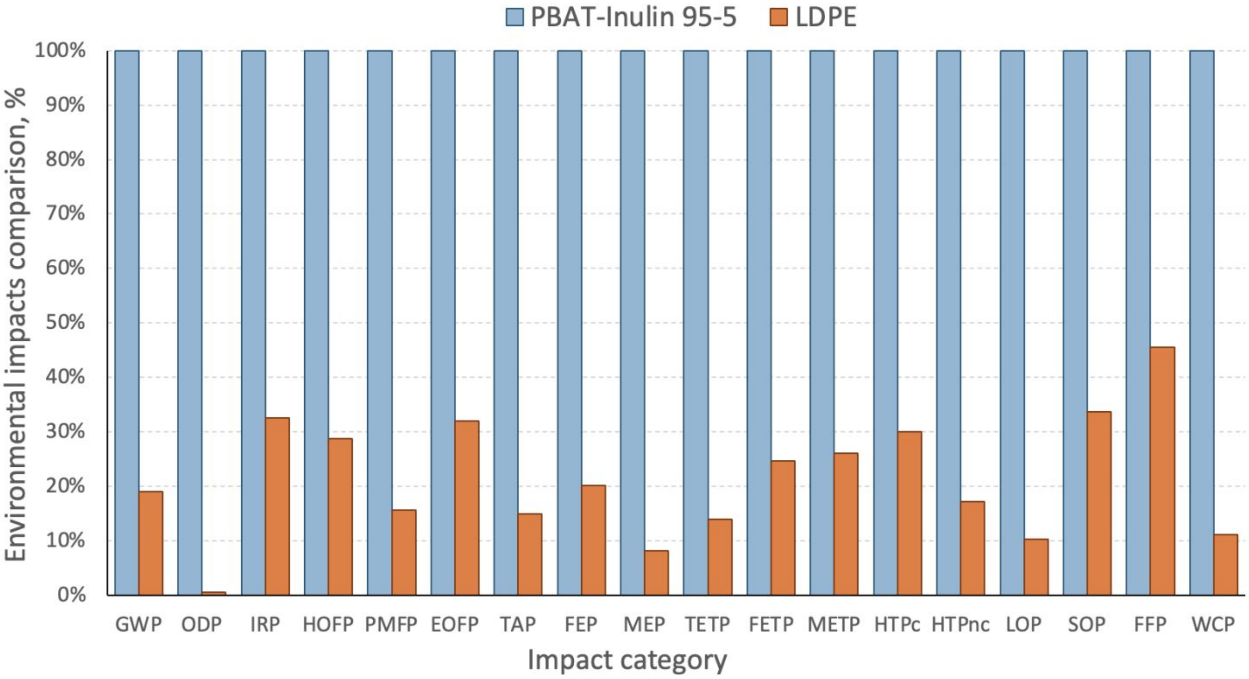

Figure S11: Comparison between the cradle-to-gate environmental impacts of the analyzed PBAT-Inulin composite and the production of LDPE, granulate; method: ReCiPe 2016 midpoint (H) v1.11/ World 2010 (H/A).

Results obtained from the comparison highlight that the commercial reference LDPE has a superior environmental profile, reporting lower impacts across all considered categories on a cradle-to-gate. However, the results for the composite were calculated at the laboratory scale, whereas those for LDPE refer to an average industrial scale in Europe, highlighting the need to reduce the potential environmental impacts of the composite during upscaling in order to improve its environmental competitiveness. Zhou et al. also compared the performance of a PBAT-montmorillonite composite film with LDPE, finding that the fossil-based polymer has a lower carbon footprint on a cradle-to-gate basis. (7)

Potential environmental benefits of biodegradable PBAT composites, compared to persistent fossil plastics, could be encountered in the end-of-life stage. (18) Traditional waste disposal methods such as landfilling and incineration would provide limited benefits for PBAT compared to commercially available non-biodegradable alternatives.

Compared to persistent fossil-based plastics, the landfilling of biodegradable plastics could entail the production of biogas, composed mostly of CH_4 and CO_2, requiring recovery to avoid greenhouse gas emissions and to valorize it for energy cogeneration. (19,20). Contrarily to bio-based materials,

in the case of PBAT, the produced CO_2 would be of fossil origin, lacking the potential benefit of net neutrality over the life cycle. Similarly, the incineration of petroleum-based PBAT would imply fossil CO_2 emissions, entailing no benefits compared to traditional plastics in terms of climate change. The integration of a bio-based fraction in the material could partially mitigate this shortcoming by reducing the emissions of fossil-based greenhouse gases. Recycling options for PBAT have also been explored in literature; e.g., Ismail et al. tested the feasibility of the enzymatic hydrolysis of pure PBAT into its monomers and the following repolymerization, on a laboratory scale setting. (21)

Due to the biodegradability of PBAT, industrial composting and anaerobic digestion are also potential viable end-of-life routes. (18) The aerobic biodegradation (composting) process of pure PBAT composting would entail the production of fossil CO_2 , water and biomass, which could be valorized as soil conditioner, resulting into environmental benefits associated with the multifunctionality of the waste management process. Similarly, the anaerobic digestion process of the biodegradable polymer would co-produce biogas and digestate, along with emissions of fossil greenhouse gases. Further testing is, anyway, still needed to assess the potential for recyclability and biodegradability of the PBAT:Inulin composite.

The integration of PBAT with bio-based inulin as a filler could mitigate potential environmental harms associated with the emissions of fossil CO_2 at the end-of-life stage of the polymer. However, a complete cradle-to-grave LCA is required to quantitatively assess the advantages and shortcomings of all possible disposal strategies, to identify potential hotspots and burden shifting across different scenarios.

Uncertainty analysis

The uncertainty associated with inventory data was evaluated by applying the Pedigree Matrix proposed by Weidema and Wesnaes, (22) assigning scores to each dataset based on the representativeness of the collected data. For the input material flows of the composite production, a score of 1 was assigned to all considered sources of uncertainty since data based on measurements were collected directly in the laboratory. Regarding electricity flows, these were calculated by multiplying the power requirements of the employed instrumentation (technical sheets) by their usage time; therefore, a score of 2 was assigned on the representativeness category, maintaining the other scores equal to 1. A basic uncertainty (Ub) of 1.05 was also assigned to all input processes, as reported in published guidance on the treatment of uncertainty in LCA. (23) Pedigree matrix relative to the production of the composite is reported in Table S17.

Table S17: Pedigree Matrix relative to the manufacture of the composite material

Composite production	Reliability	Completeness	Temporal correlation	Geographical correlation	Further technological correlation
Material flows	1	1	1	1	1
Electricity flows	2	1	1	1	1

The inventories of the production of bio-based precursors were modelled based on literature results of mass and energy balance calculations and software simulations, rather than industry data from technologies deployed on a market scale. As such, scores above 1 were assigned on all categories to account for the higher uncertainty relative to the representativeness of the modelled inventories. Pedigree Matrix is reported in Table S18.

Table S18: Pedigree Matrix relative to the production of PBAT monomers

PBAT monomers	Reliability	Completeness	Temporal correlation	Geographical correlation	Further technological correlation
Input flows, 1,4-BDO	2	4	3	3	4
Input flows, bio-AA	2	4	2	5	4
Input flows, bio-TA	2	4	2	5	4

Regarding the polymerization process of PBAT, the same uncertainty scores assigned by default to the ecoinvent process for PET were maintained.

For the production of inulin, data relative to its extraction were based on Hinghsamer et al, (14)reporting an LCA based on industrial data from a dedicated plant; hence, data were deemed as representative from a technological standpoint. The organic and inorganic emissions to water were assigned a Ub equal to 1.50. Scores are reported in Table S19.

Table S19: Pedigree Matrix relative to the production of inulin

Inulin production	Reliability	Completeness	Temporal correlation	Geographical correlation	Further technological correlation
Input flows, Inulin	2	3	1	1	1

Results of the Monte Carlo analysis (1000 runs) conducted to compare fossil-PBAT_S with 50%PV + bio-PBAT_S are reported in Table S20.

Table S20: Monte Carlo analysis results conducted to compare fossil-PBAT_S (A) to 50%PV + bio-PBAT_S (B); method: ReCiPe 2016 endpoint (H) v1.11/ World 2010 (H/A).

Damage category	A >= B	Mean	Median	SD	CV	2.5%	97.5%	SEM
Ecosystems	3.7 %	-3.02E-08	-2.84E-08	2.3E-08	-76.2 %	-6.97E-08	3.91E-09	7.28E-10
Human health	50.8 %	-3.76E-06	0.000113	0.00525	-1.4E5 %	-0.0111	0.0104	0.000166
Resources	99.7 %	0.37	0.363	0.148	40 %	0.105	0.702	0.00469

References

(1) Wang BX, Cortes-Peña Y, Grady BP, Huber GW, Zavala VM. Techno-Economic Analysis and Life Cycle Assessment of the Production of Biodegradable Polyaliphatic-Polyaromatic Polyesters. ACS Sustain Chem Eng. 2024 Jun 17;12(24):9156–67. https://doi.org/10.1021/acssuschemeng.4c01842

(2) Ratshoshi BK, Farzad S, Görgens JF. A techno-economic study of Polybutylene adipate terephthalate (PBAT) production from molasses in an integrated sugarcane biorefinery. Food and Bioproducts Processing. 2024 May 1;145:11–20. https://doi.org/10.1016/j.fbp.2024.01.011

(3) Luo C, Zhou Y, Chen Z, Bian X, Chen N, Li J, et al. Comparative life cycle assessment of PBAT from fossil-based and second-generation generation bio-based feedstocks. Science of the Total Environment. 2024 Dec 1;954. https://doi.org/10.1016/j.scitotenv.2024.176421

(4) Hernandez-Charpak YD, Mozrall AM, Williams NJ, Trabold TA, Diaz CA. Biochar as a sustainable alternative to carbon black in agricultural mulch films. Environ Res. 2024 Apr 1;246. https://doi.org/10.1016/j.envres.2023.117916

(5) Akbarian-Saravi N, Larizadeh R, Gupta A, Shum D, Milani AS. Life Cycle Assessment Sheds New Insights Toward Sustainable Management of Biodegradable Resin Blends Used in Packaging: A Case Study on PBAT. Sustainability. 2025 Sep 25;17(19):8645. https://doi.org/10.3390/su17198645

(6) Hasan Y, Abbassi B, Mohanty AK, Misra M, Bali A, Tiessen M. Life cycle assessment of poly (butylene adipate-co-terephthalate) (PBAT)-talc Ontario agri-film. Cleaner Environmental Systems. 2025 Dec 1;19. https://doi.org/10.1016/j.cesys.2025.100360

(7) Zhou SJ, Zhang D, Xiong SJ, Liu Q, Shen X, Yu S, et al. A High-Performance and Cost-Effective PBAT/Montmorillonite/Lignin Ternary Composite Film for Sustainable Production. ACS Sustain Chem Eng. 2024 Oct 7;12(40):14704–15. https://doi.org/10.1039/D4EN01197J

(8) Abdelwahab MA, Rabnawaz M, Khan A, Shaker M, Elkholy HM, Bher A, et al. Chemically and mechanically recyclable polyester-based multilayer plastics. Chemical Engineering Journal.

2025 Dec 1;525. https://doi.org/10.1016/j.cej.2025.169956

(9) Volanti M, Cespi D, Passarini F, Neri E, Cavani F, Mizsey P, et al. Terephthalic acid from renewable sources: Early-stage sustainability analysis of a bio-PET precursor. Green Chemistry. 2019;21(4):885–96. https://doi.org/10.1039/C8GC03666G

(10) Forte A, Zucaro A, Basosi R, Fierro A. LCA of 1,4-butanediol produced via direct fermentation of sugars from wheat straw feedstock within a territorial biorefinery. Materials. 2016;9(7). https://doi.org/10.3390/ma9070563

(11) Corona A, Biddy MJ, Vardon DR, Birkved M, Hauschild MZ, Beckham GT. Life cycle assessment of adipic acid production from lignin. Green Chemistry. 2018;20(16):3857–66. https://doi.org/10.1039/C8GC00868J

(12) International Energy Agency, 2023. Italy. Available at https://www.iea.org/countries/italy/energy-mix. (accessed on: 29/06/2025)

(13) Schrijvers DL, Leroux F, Verney V, Patel MK. Ex-ante life cycle assessment of polymer nanocomposites using organo-modified layered double hydroxides for potential application in agricultural films. Green Chemistry. 2014 Dec 1;16(12):4969–84. https://doi.org/10.1039/C4GC00830H

(14) Hingsamer M, Kulmer V, de Roode M, Kernitzkyi M. Environmental and socio-economic impacts of new plant breeding technologies: A case study of root chicory for inulin production. Front Genome Ed. 2022;4. https://doi.org/10.3389/fgeed.2022.919392

(15) Unlu S, Niu W, Demirel Y ar. Bio-based adipic acid production: feasibility analysis using a multi-criteria decision matrix. Biofuels, Bioproducts and Biorefining. 2020 Jul 1;14(4):794–807. https://doi.org/10.1002/bbb.2106

(16) Vardon DR, Rorrer NA, Salvachúa D, Settle AE, Johnson CW, Menart MJ, et al. Cis, cis - Muconic acid: Separation and catalysis to bio-adipic acid for nylon-6,6 polymerization. Green Chemistry. 2016;18(11):3397–413. https://doi.org/10.1039/C5GC02844B

(17) Blonk, H., Tyszler, M., van Paassen, M., Braconi, N., Draijer, N., van Rijn, J., 2022. Agri-footprint 6 Methodology Report. Available at: https://blonksustainability.nl/agri-footprint#gsc.tab=0 (Accessed on 03/07/2025).

(18) Van Roijen EC, Miller SA. A review of bioplastics at end-of-life: Linking experimental biodegradation studies and life cycle impact assessments. Vol. 181, Resources, Conservation and Recycling. Elsevier B.V.; 2022. https://doi.org/10.1016/j.resconrec.2022.106236

(19) Rodrigo-Ilarri J, Rodrigo-Clavero ME. Mathematical modeling of the biogas production in MSW landfills. Impact of the implementation of organic matter and food waste selective collection systems. Atmosphere (Basel). 2020;11(12):1–18. https://doi.org/10.3390/atmos11121306

(20) Hobbs SR, Harris TM, Barr WJ, Landis AE. Life cycle assessment of bioplastics and food waste disposal methods. Sustainability (Switzerland). 2021 Jun 2;13(12). https://doi.org/10.3390/su13126894

(21) Ismail M, Abouhmad A, Warlin N, Pyo SH, Örn OE, Al-Rudainy B, et al. Closing the loop for poly(butylene-adipate-co-terephthalate) recycling: depolymerization, monomers separation, and upcycling. Green Chemistry. 2024 Feb 21;26(7):3863–73. https://doi.org/10.1039/D3GC04728H

(22) Pedersen Weidema B, Suhr Wesnaes M. Data quality management for life cycle inventories-an example of using data quality indicators*. Vol. 4, J. Cleaner Prod. 1996. https://doi.org/10.1016/S0959-6526(96)00043-1.

(23) Goedkoop M, Oele M, Leijting J, Ponsioen T, Meijer E. Introduction to LCA with SimaPro, 2016. Available from: www.pre-sustainability.com
